# Optical see-through augmented reality via inverse-designed waveguide couplers

**DOI:** 10.1515/nanoph-2025-0501

**Published:** 2025-12-08

**Authors:** Seunghyun Lee, Byounghyo Lee, Haejun Chung

**Affiliations:** Department of Electronic Engineering, 26716 Hanyang University, Seoul, 04763, South Korea; Korea Electronics Technology Institute, Seoul, 03924, South Korea; Department of Electronic Engineering, and Department of Artificial Intelligence, 26716 Hanyang University, Seoul, 04763, South Korea

**Keywords:** metasurface, waveguide couplers, augmented reality waveguides, inverse design, see-through view

## Abstract

Waveguide-based augmented-reality (AR) displays offer compact, optical see-through form factors but remain limited by chromatic dispersion, ghosting from parasitic diffraction orders, distortion of the see-through scene, and a restricted eyebox. We present triple-function metasurface couplers designed using adjoint-based optimization, which overcome these limitations and establish computational performance bounds. The out-coupler simultaneously preserves zeroth-order transmission of the see-through path and directs display light into designated diffraction orders while returning residual guided power as zeroth-order reflection for eyebox expansion. The in-coupler assigns distinct diffraction orders to R/G/B and equalizes their in-plane propagation angles, achieving achromatic guidance and eliminating chromatic path divergence. Quantitatively, the optimized out-coupler provides >90 % angle-averaged zeroth-order transmission for the see-through view (10–40× lower higher-order leakage) and >95 % zeroth-order guided reflection, while maintaining efficient diffractive couplings to the eyebox. PSF/MTF analyses confirm near-diffraction-limited virtual-image quality and strong suppression of see-through view distortion. Finally, benchmarking freeform against fabrication-constrained multilayer architectures (1–6 layers) shows that multilayers approach the freeform upper bound while remaining practical to fabricate. These results outline a general, manufacturable methodology for multifunctional metasurface couplers and a practical route to compact, high-quality AR waveguides.

## 1 Introduction

Augmented reality (AR) is an emerging display technology that allows virtual images to be overlaid onto the user’s view of the physical world in real time. Since its conceptual origin in the late 1960s [1], AR has undergone significant evolution, progressing from rudimentary head-mounted displays to sophisticated systems capable of dynamic context-aware visualization. These developments have broadened the application landscape of AR – extending to education by supporting interactive visualization and laboratory practice [2], to manufacturing by providing in-view procedural guidance and remote expert collaboration [3], to healthcare by delivering image-guided navigation and therapy monitoring [4], and to entertainment by enabling location-based applications and immersive narrative experiences [5]. To enable such experiences, various optical architectures have been explored, including birdbath optics [6], freeform optics [7], [8], [9], pin-mirror arrays [10], [11], [12], and Maxwellian view displays [13], [14], [15]. However, many of these optical architectures suffer from bulky components, limited eyebox sizes, poor see-through performance, and image artifacts such as ghosting, optical distortion, or alignment.

To address the growing demands for compactness, lightweight design, and visual unobtrusiveness in AR devices, waveguide-based optical architectures have emerged as a leading solution [16], [17], [18]. Unlike conventional systems, waveguide displays guide light within a thin, transparent substrate via total internal reflection (TIR), enabling eyeglass-like form factors while supporting both a wide field of view (FoV) and an enlarged eyebox. In diffractive waveguide combiners, surface-relief gratings (SRG) [19] or volume holographic gratings (VHG) [20], [21] located at the input and output regions enable efficient coupling, injecting image-bearing light into guided modes and extracting it toward the eye. These attributes have motivated the adoption of waveguide optics in commercial AR products [22], [23], [24].

Despite their advantages, conventional SRG/VHG waveguide-based AR devices exhibit several critical limitations [25], [26], [27]. The periodic diffractive couplers inherently cause spectral spread and chromatic dispersion, leading to wavelength-dependent angular shifts that constrain the attainable full-color FoV and yield color nonuniformity across the eyebox [28]. Higher-order diffraction and leakage into unintended orders generate ghost images and reduce image contrast [29]. To mitigate dispersion, many products adopt a stack of multiple waveguides with separate guides dedicated to the red, green, and blue channels. Although this architecture reduces chromatic error, it increases the overall thickness of the optical combiner and substantially raises fabrication complexity [30], [31].

To address these limitations, metasurface-based grating couplers have emerged as a promising alternative. Leveraging subwavelength-scale structural engineering, metasurfaces offer unprecedented control over the phase, amplitude, and polarization of light, enabling highly customized diffraction responses within ultrathin form factors [32], [33], [34], [35], [36]. Moreover, metasurfaces can be designed to operate over broad spectral ranges, enabling their application in diverse optical functionalities such as flat lenses [37], [38], [39], [40], beam shaping [41], [42], polarization conversion [43], beam splitting [44], [45], and beam steering [46], [47]. Their planar geometry and compatibility with existing semiconductor fabrication processes make them particularly attractive for integration into AR waveguides.

Several recent studies have explored metasurface-based coupler designs to improve the performance of AR waveguide displays. A recent study [48] demonstrated a dielectric metasurface coupler optimized for uniform diffraction efficiencies across RGB wavelengths. The work of Liang et al. [49] introduced a hybrid topology optimization combining a simulated annealing algorithm to design unpolarized freeform in-couplers for AR waveguides. Sun et al. [50] proposed a compound metasurface approach that combines a double-layer grating structure with cylindrical phase elements, enabling highly efficient achromatic light coupling. Gopakumar et al. [51] realized compact full-color 3D holography in AR by integrating inverse-designed metasurface gratings and AI-driven holographic reconstruction. While these approaches have demonstrated meaningful advances in metasurface-based waveguide coupling, several practical considerations for AR display deployment remain insufficiently addressed. In particular, the optimization of the eyebox, the spatial region within which the user’s eye can perceive the virtual image, is rarely considered. In addition, the diffraction of external ambient light that enters the waveguide, often neglected in metasurface coupler designs, can distort the appearance of the real-world scene, thereby degrading the overall AR experience.

Furthermore, when the same diffraction order is employed for guided modes across the RGB wavelengths, each color typically follows a distinct propagation path within the waveguide. This effect induces spatial dispersion, leading to image misalignment at the output. To overcome this issue, recent studies [52], [53] proposed an achromatic metasurface coupler that assigns different diffraction orders to the RGB wavelengths while maintaining identical diffraction angles, thereby ensuring aligned propagation within the waveguide. Although this approach effectively suppresses spatial dispersion, the out-coupler design remains suboptimal in managing reflective diffraction orders, thereby limiting its ability to enhance eyebox performance. Moreover, no prior studies have demonstrated simultaneous control of both guided light and see-through scene at the out-coupler design for directing them into the desired diffraction orders.

To overcome this issue, recent studies proposed achromatic metasurface couplers that assign different diffraction orders to the RGB wavelengths while maintaining identical diffraction angles, thereby ensuring aligned propagation within the waveguide. Although this approach effectively suppresses spatial dispersion, the out-coupler design remains limited in the comprehensive management of reflective diffraction orders. In particular, while Moon et al. [52] incorporated reflective-order control, prior works have not demonstrated simultaneous optimization of both the transmitted and reflected fields of guided light together with ambient light at the out-coupler, nor shown directing all of them into desired diffraction orders.

**Figure 1:**
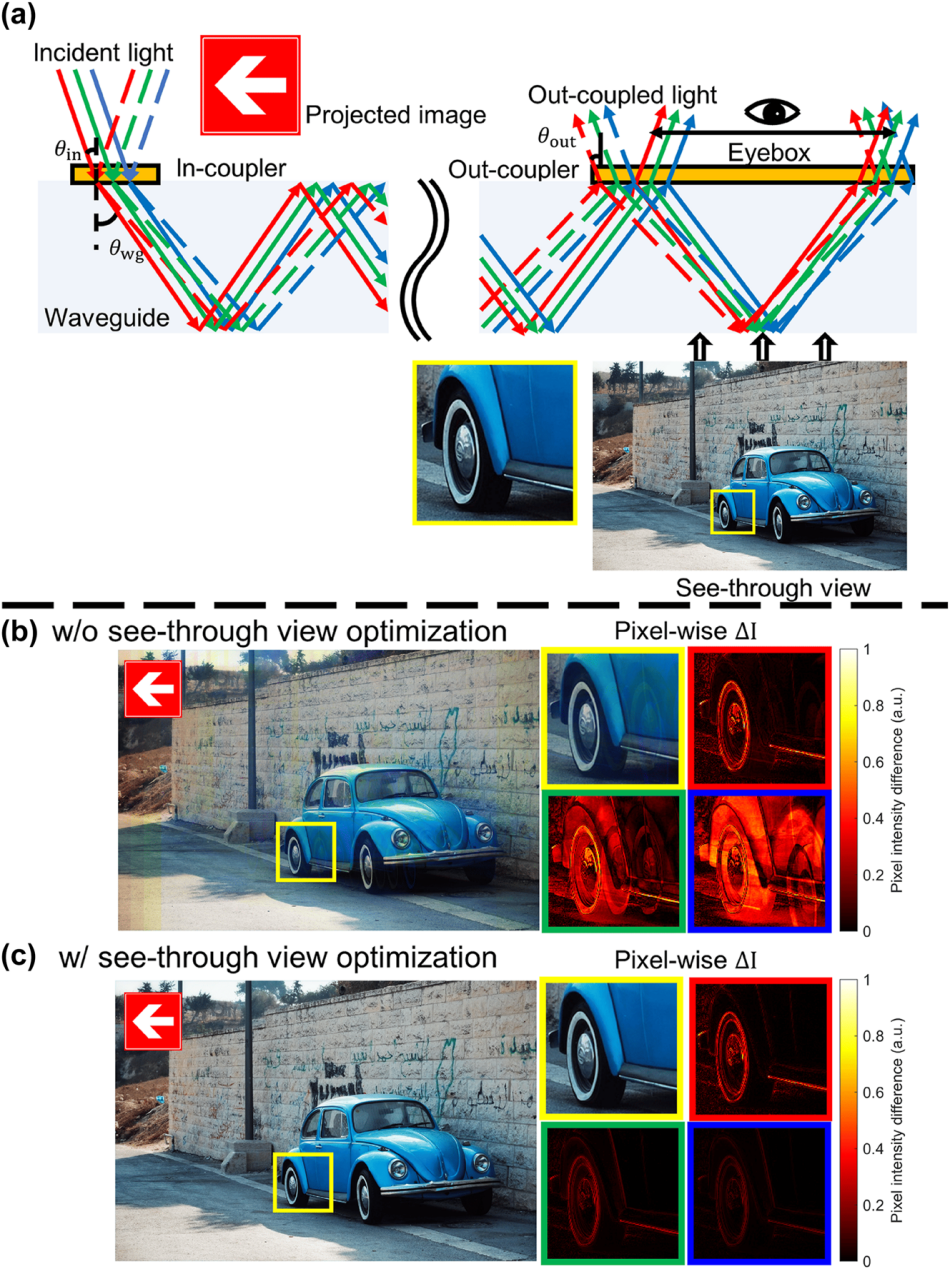
Schematic of the proposed metasurface-waveguide AR system and the resulting see-through performance. (a) Optical diffraction control with the triple-function design. The red arrow symbolizes the projected virtual image being directed toward the eyebox. The in-coupler assigns distinct diffraction orders to RGB while equalizing their in-plane propagation angle, and the out-coupler sends guided power to the designated transmission orders, returns the remainder as zeroth-order guided reflection for eyebox expansion, and passes normally incident ambient light in the zeroth order to preserve the see-through view. (b) Design without see-through view optimization: normally incident ambient light is redistributed into higher diffraction orders, producing blur, ghost artifacts. The pixel-wise Δ*I* plots show the intensity error relative to the original scene, visualized separately for the RGB channels. (c) Design with see-through view optimization: higher-order leakage is suppressed, the pixel-wise intensity error is reduced, and the see-through view closely matches the original scene.

In this work, we introduce multifunctional metasurface couplers that address key limitations of AR waveguides. The in-coupler is independently optimized to route the red, green, and blue channels into distinct diffraction orders while equalizing their in-plane propagation angles and balancing order efficiencies, thereby achieving achromatic in-plane guidance. The out-coupler is optimized to perform its triple-function role by simultaneously: (i) diffracting guided image light into designated orders to form the eyebox; (ii) suppressing spurious diffraction of the transmitted see-through view to reduce blur and ghost artifacts and improve visual clarity, and (iii) returning residual guided power as zeroth-order reflection that remains trapped by total internal reflection, enabling repeated encounters with the out-coupler and efficient multi-pass extraction with minimal loss. Without the see-through objective, incident ambient light is redistributed into higher diffraction orders, producing blur, ghosting, and large pixel-wise intensity differences relative to the ground truth [Figure 1(b)]; with the objective included, higher-order leakage is strongly suppressed, the pixel-wise difference is minimized, and the see-through view closely matches the original scene [Figure 1(c)].

We realize this multifunctionality using an adjoint-based inverse-design framework [54], [55] that enables efficient gradient computation and scalable optimization of both in- and out-coupler geometries. We analyze two distinct architectures: freeform metasurfaces to establish computational performance upper bounds, and fabrication-constrained multilayer metasurfaces. This multilayer approach is specifically investigated to provide the additional degrees of freedom necessary to overcome the efficiency and purity limitations inherent in simple single-layer gratings, bridging the gap between theoretical bounds and lithographically practical implementations.

Imaging performance under realistic viewing conditions is quantified via point-spread-function and modulation-transfer-function analyses, which show substantial suppression of ghosting and see-through distortion [Figure 1(b) and (c)], paving the way toward compact, high-quality AR waveguide systems.

## 2 Design principles and optimization frameworks

In this section, we establish the theoretical and computational framework for designing metasurface-based couplers for AR waveguides. We first introduce the concept of diffraction order assignments for chromatic alignment, which enables all RGB channels to share a common in-plane propagation angle inside the waveguide. Next, we describe the plane-wave decomposition method used to quantify angular and spectral responses in terms of discrete diffraction orders. Finally, we present the adjoint-based optimization framework that integrates plane-wave decomposition into the FoM, allowing efficient inverse design of both in-couplers and out-couplers under practical fabrication constraints.

### 2.1 Diffraction order control for chromatic alignment

A major challenge in metasurface-based waveguide coupling for AR displays is achieving both high diffraction efficiency and achromatic performance across RGB wavelengths. In conventional grating-based designs, a single diffraction order is applied to all wavelengths. Due to the wavelength dependence of the grating equation, each color couples into the waveguide at a different angle, producing angular dispersion and divergent propagation paths. This angular mismatch results in chromatic separation within the waveguide, leading to spatial misalignment at the out-coupler and degraded image quality.

Furthermore, the range of allowable guided angles is limited by the TIR condition. For broadband operation, the grating period must be small enough for the longest wavelength to remain within the guided range. Since longer wavelengths diffract at larger angles, this constraint forces a shorter period, which in turn reduces the maximum achievable coupling angle for shorter wavelengths. The result is a narrower angular acceptance window and thus a reduced FoV.

**Figure 2:**
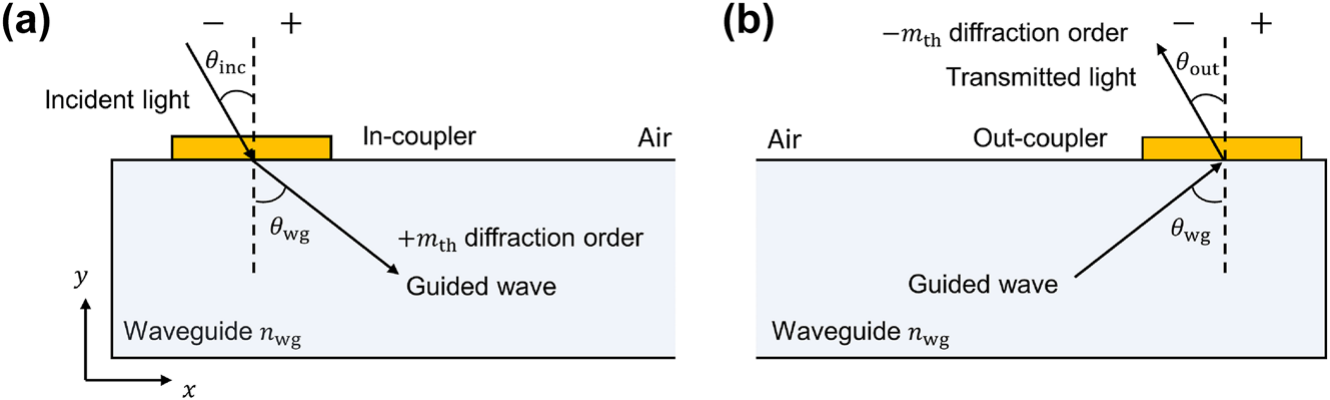
Diffraction-order assignment for achromatic guidance in AR waveguides. (a) In-coupling: an incident beam at angle *θ*_inc_ is diffracted by the in-coupler into the +*m*-th order, launching a guided mode at *θ*_wg_ inside a waveguide of index *n*_wg_. (b) Out-coupling: the guided mode is extracted by the out-coupler using the opposite order −*m*, emitting to air at *θ*_out_. Assigning different diffraction orders to R/G/B such that *m*_R_ *λ*_R_ = *m*_G_ *λ*_G_ = *m*_B_ *λ*_B_ yields a common in-plane propagation angle and eliminates angular chromatic dispersion while preserving spatial color alignment.

To overcome these limitations, we assign a distinct diffraction order to each wavelength so that all colors couple into the waveguide at the same in-plane propagation angle. The in-coupling process, shown in Figure 2(a), follows

(1) $$n_{\text{wg}}\sin\theta_{\text{wg}}+n_{\text{air}}\sin\theta_{\text{inc}}=m\frac{\lambda }{\Lambda_{x}},$$

where *n*_air_ = 1, *n*_wg_ = 2.0 (high-index glass waveguide), *λ* is the wavelength, Λ_*x*_ is the grating period, and *m* is the diffraction order. By choosing *m*_R_ = +4 for red (*λ*_R_ = 675 nm), *m*_G_ = +5 for green (*λ*_G_ = 540 nm), and *m*_B_ = +6 for blue (*λ*_B_ = 450 nm), we satisfy

(2) $$m_{\text{R}}\lambda_{\text{R}}=m_{\text{G}}\lambda_{\text{G}}=m_{\text{B}}\lambda_{\text{B}},$$

ensuring that *θ*_wg_ is identical for all colors. This common angle is selected to lie within the TIR range $\left(\theta_{\text{c}}=30{}^{\circ}<\theta_{\text{wg}}<60{}^{\circ}\right)$ , avoiding leakage while maintaining symmetric FoV coverage.

For chromatic alignment at the output as depicted in Figure 2(b), the out-coupler applies the opposite diffraction orders (−4, −5, and −6 for R, G, and B, respectively). Its operation is governed by

(3) $$n_{\text{wg}}\sin\theta_{\text{wg}}+n_{\text{air}}\sin\theta_{\text{out}}=m^{\prime }\frac{\lambda }{\Lambda_{x}},$$

where *θ*_out_ is the desired emission angle (e.g., 0° for normal output) and *m*′ is the out-coupler diffraction order. Setting *m*′ = −*m* ensures that each wavelength exits at the same free-space angle, eliminating angular chromatic aberrations. This opposite-order configuration reverses the in-plane wavevector. As a result, the wavefront is reconstructed at the output without angular distortion, and spatial color registration is maintained.

### 2.2 Planewave decomposition

To characterize the angular and spectral response of the metasurface, we employ a plane-wave decomposition framework in which the transmitted field is expressed as a superposition of discrete diffraction orders. For a periodic metasurface illuminated by a plane wave, the transmitted field can be expanded as

(4) $$E_{\text{trans}}\left(r\right)=\underset{m,n,\sigma }{\sum }C_{m,n,\sigma }\;{\hat{\varepsilon }}_{m,n,\sigma }\;{\text{e}}^{\text{i}k_{m,n}\cdot r},$$

where *C*_*m*,*n*,*σ*_ is the complex amplitude of the (*m*, *n*)-th diffraction order with polarization *σ*, ${\hat{\varepsilon }}_{m,n,\sigma }$ is the corresponding unit polarization vector, and **k**_*m*,*n*_ is the wavevector of that order.

For a metasurface with periodicities Λ_*x*_ and Λ_*y*_ along the *x*- and *y*-axes, respectively, the transmitted electric field can be expanded as:

(5) $$k_{m,x}=k_{x}+m\frac{2\pi }{\Lambda_{x}},\;k_{n,y}=k_{y}+n\frac{2\pi }{\Lambda_{y}}.$$

The *z* component of the wavevector in the transmission medium is obtained from the dispersion relation:

(6) $$k_{m,n,z}=\sqrt{\epsilon_{r}{\left(\frac{\omega }{c}\right)}^{2}-k_{m,x}^{2}-k_{n,y}^{2}},$$

where *ϵ*_*r*_ is the relative permittivity of the output medium. Modes with real *k*_*m*,*n*,*z*_ are propagating, while those with imaginary values are evanescent and do not contribute to the far field.

The amplitude of each diffraction mode is extracted by projecting the transmitted field onto the corresponding plane-wave basis:

(7) $$A_{m,\sigma }=\frac{1}{A}\underset{S}{\int }E_{\text{trans}}\left(r\right)\cdot {\hat{\varepsilon }}_{m,\sigma }^{*}\;{\text{e}}^{-\text{i}k_{m}\cdot r}\;dA,$$

where *S* is the integration surface in the transmission region and *A* is its area. The orthogonality of plane waves ensures that only components matching the target mode contribute to *A*_*m*,*σ*_.

The power carried by each propagating diffraction order is computed from the time-averaged Poynting vector as

(8) $$P_{m,\sigma }=\frac{A}{2Z_{t}}\;|A_{m,\sigma }{|}^{2}\;\text{Re}\;\left(\frac{k_{m,z}}{\left|k_{m}\right|}\right),$$

where $Z_{t}=\sqrt{\mu /\epsilon }$ is the wave impedance of the transmission medium.

### 2.3 Adjoint optimization

Conventional global optimization techniques, including genetic algorithms [56], [57] and particle swarm optimization [58], often demand a prohibitively large number of full-wave simulations to adequately sample the design space, resulting in high computational cost and slow convergence. Semi-analytical approaches such as rigorous coupled-wave analysis [59] offer faster evaluations but are typically constrained to simple, idealized geometries, making them unsuitable for complex freeform metasurfaces. Data-driven strategies, such as machine learning-based inverse design [60], [61], [62], can accelerate the search for optimal solutions; however, they generally require substantial training datasets of high fidelity, limiting their applicability to untrained parameter regimes.

Adjoint-based optimization provides a more scalable alternative by enabling the calculation of the gradient of FoM with respect to all design variables from only two full-wave simulations: a forward run and an adjoint run. This makes it highly efficient for large-scale freeform designs, where the number of variables can reach millions and direct parameter sweeps become impractical.

In this work, we integrate adjoint optimization with a planewave decomposition framework, allowing the FoM to be explicitly defined in terms of the power in specific diffraction orders. This integration allows the optimization to direct more power into the designated diffraction orders for AR waveguide coupling, while simultaneously minimizing leakage into non-target orders.

**Figure 3:**
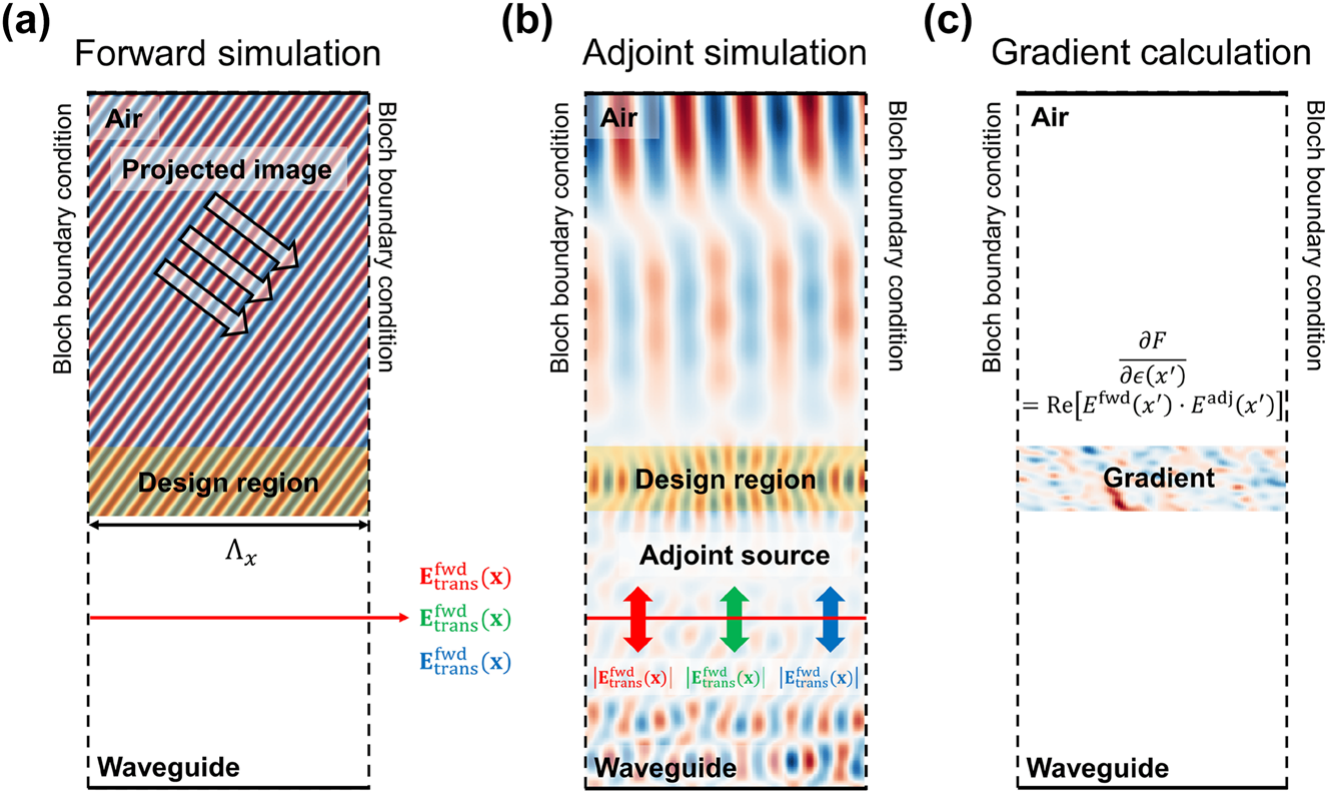
Adjoint-based optimization framework for metasurface in-couplers. (a) Forward simulation: incident plane waves at different angles are launched from air, and the transmitted fields into the waveguide are monitored and decomposed into diffraction orders. (b) Adjoint simulation: sources are placed at the monitor with field distribution determined from the forward fields and the FoM derivative, back-propagating through the design region. (c) Gradient calculation: the overlap of forward and adjoint fields yields the spatial sensitivity with respect to the local permittivity distribution, which is accumulated over wavelengths and incidence angles to update the design.

Figure 3 depicts the adjoint workflow used for the in-coupler. In the forward run shown in Figure 3(a), plane waves incident from air at a discrete set of incidence angles {*ϕ*_*s*_} spanning the FoV are simulated. For each wavelength-angle sample (*λ*, *ϕ*_*s*_), the complex field transmitted into the waveguide is sampled on a monitor placed immediately beneath the metasurface and decomposed into diffraction orders to obtain the per-order powers *P*_*m*_(*λ*, *ϕ*_*s*_) as described in Section 3.1. In the adjoint run in Figure 3(b), a source located at the same monitor has amplitude

(9) $$J_{\text{adj}}\left(x\right)=-i\omega \;\frac{\partial F}{\partial E\left(x\right)},$$

where $\frac{\partial F}{\partial E\left(x\right)}$ denotes the functional derivative of the FoM with respect to the complex electric field evaluated at the monitor. This source back-propagates through the structure, generating the adjoint fields **E** ^adj^(**x**; *λ*, *ϕ*_*s*_). The spatial gradient is then obtained from the forward-adjoint overlap [Figure 3(c)],

(10) $$\frac{\partial F}{\partial \epsilon \left(x\right)}=\text{Re}\;\;\left[E^{\text{fwd}}\left(x\right)\cdot E^{\text{adj}}\left(x\right)\right],$$

and the gradients are accumulated over all wavelength-angle samples using adaptive weights to promote uniform performance across wavelengths and FoV.

**Figure 4:**
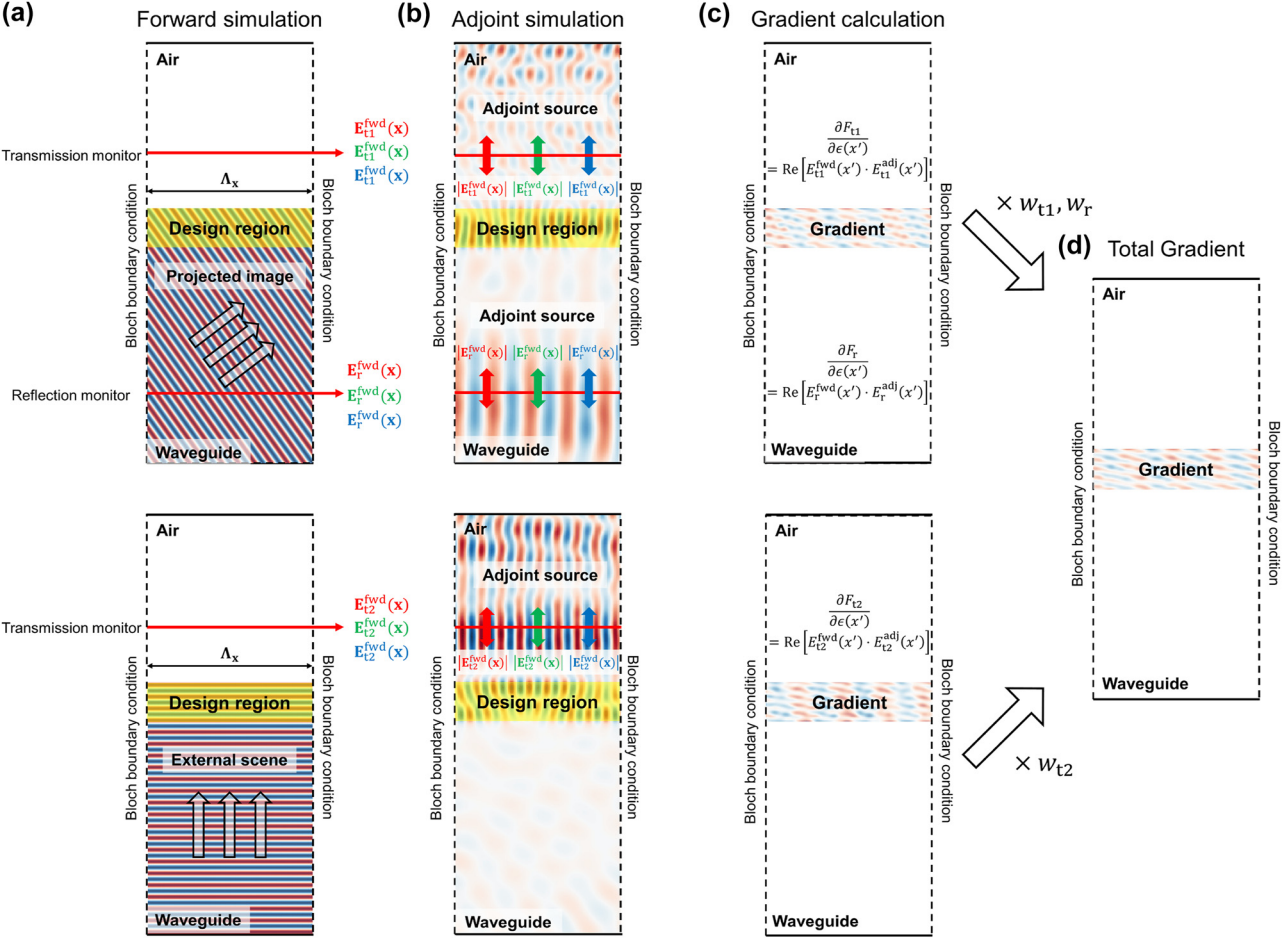
Adjoint-based optimization framework for metasurface out-couplers. (a) Forward simulations: guided waves spanning in-plane propagation angles and normally incident plane waves representing ambient light are injected. The transmitted and reflected fields are recorded at monitor planes and decomposed into diffraction orders. (b) Adjoint simulations: sources are placed at the same monitors, with field distributions defined by the derivative of each FoM with respect to the complex fields. (c) Gradient calculation: local gradients are computed from the overlap of forward and adjoint fields for each FoM, including reflection (*F*_*r*_), guided-wave transmission (*F*_*t*1_), and suppression of unwanted diffraction of external light (*F*_*t*2_). (d) The weighted sum of individual gradients produces the total gradient for updating the design region in each iteration.

In the forward simulation for the out-coupler as shown in Figure 4(a), two excitation cases are considered. First, guided waves spanning a continuous range of in-plane propagation angles from the in-coupler are discretely sampled and coupled into the metasurface at oblique incidence, enabling characterization of the angular dependence of diffraction. Second, a normally incident plane wave is introduced to represent the see-through scene, allowing assessment of the out-coupler’s ability to suppress unwanted diffraction. For the guided-wave case, both transmitted and reflected fields are obtained at the designated monitor planes, whereas for the see-through view case, only transmitted fields are acquired. In each case, the captured fields are decomposed into discrete diffraction orders via a planewave decomposition, providing the complex amplitudes and powers used for subsequent FoM evaluation.

In the adjoint simulation as illustrated in Figure 4(b), sources are placed at the same monitor planes where the FoMs are defined, and their profiles are determined directly from the forward-simulated fields according to the FoM being optimized. Three FoMs are considered in total: the reflection FoM for guided waves (*F*_*r*_), the transmission FoM for guided waves (*F*_*t*1_), and the transmission FoM for external light (*F*_*t*2_). The index *j* ∈ {*r*, *t*1, *t*2} is used to refer to each FoM in the following formulation of the adjoint source:

(11) $$J_{\text{adj},j}\left(x\right)=-i\omega \frac{\partial F_{j}}{\partial E_{\text{j}}\left(x\right)},$$

where $\frac{\partial F_{j}}{\partial E_{\text{j}}\left(x\right)}$ denotes the functional derivative of the *j*-th FoM with respect to the complex electric field at the corresponding monitor plane. This formulation guarantees that the backward-propagated fields generated in the adjoint simulation directly correspond to the physical effect being optimized for each FoM.

As shown in Figure 4(c), the gradient for each FoM *F*_*j*_ with respect to the local permittivity is obtained from the overlap integral between the corresponding adjoint fields $E_{j}^{\text{adj}}$ and the forward fields $E_{j}^{\text{fwd}}$ :

(12) $$\frac{\partial F_{j}}{\partial \epsilon_{i}\left(x\right)}=\text{Re}\;\;\left[E_{j}^{\text{fwd}}\left(x\right)\cdot E_{j}^{\text{adj}}\left(x\right)\right],$$

which has the same form as the in-coupler case, with the difference lying only in the definition of the FoM and the corresponding source-monitor configuration.

As illustrated in Figure 4(d) the total gradient for updating the design region is then computed as a weighted sum of the individual FoM gradients:

(13) $$\frac{\partial F_{\text{total}}}{\partial \epsilon_{i}}=w_{r}\frac{\partial F_{\text{r}}}{\partial \epsilon_{i}}+w_{t1}\frac{\partial F_{\text{t}1}}{\partial \epsilon_{i}}+w_{t2}\frac{\partial F_{\text{t}2}}{\partial \epsilon_{i}},$$

where *w*_*r*_, *w*_*t*1_, and *w*_*t*2_ are the weighting factors assigned to each FoM. The computed total gradient in Eq. (13) is used to iteratively update the design variables via the Adam optimization algorithm [63], which adaptively adjusts the step size for each variable based on the first and second moments of the gradient. This approach improves convergence stability and efficiency, particularly for large-scale photonic inverse-design problems.

The optimization operates on normalized design variables *v*_*i*_ ∈ [0, 1], where *v*_*i*_ = 0 and *v*_*i*_ = 1 map to the two constituent dielectrics. These values do not represent the actual physical permittivity directly, but instead act as interpolation weights between the two materials. To impose a minimum feature size, we smooth the material density field *ρ* using a linear convolution:

(14) $$v^{\prime }\left(x\right)=\omega \left(x\right)*v\left(x\right),$$

where *v*′ is the filtered density, *ω* is a conic kernel, and ∗ denotes multidimensional convolution [64]. The conic kernel with radius *r*_min_ acts as a spatial low-pass filter that suppresses features and gaps smaller than *r*_min_, effectively enforcing a minimum feature size on the order of *r*_min_. To promote binarization, we apply a widely used Tanh projection filter [65]:

(15) $${\tilde{v}}_{i}^{\prime }=\frac{\tanh\left(\beta \eta \right)+\tanh\;\left(\beta \left(v_{i}^{\prime }-\eta \right)\right)}{\tanh\left(\beta \eta \right)+\tanh\;\left(\beta \left(1-\eta \right)\right)},$$

where ${\tilde{v}}_{i}$ denotes the projected design variable for the *i*-th pixel, and *β* and *η* are the projection parameters: *β* controls the steepness and *η* is the threshold. A continuation scheme gradually increases *β* from a small to a large value, enabling a smooth transition from grayscale to binary during optimization. The design variables *v*_*i*_ are updated each iteration using the Adam algorithm, with gradients computed via the chain rule through the smoothing and projection steps.

## 3 In-coupler design and optimization

The in-coupler serves as the optical interface that couples free-space, image-bearing light into guided modes of the AR waveguide. The diffraction characteristics of the in-coupler directly determine the coupling efficiency, the range of incident angles that can be accepted, and the chromatic alignment in guided propagation. In this work, the in-coupler is implemented as a dielectric metasurface designed to diffract each RGB wavelength into a distinct diffraction order, thereby ensuring a common in-plane propagation direction and eliminating chromatic dispersion within the waveguide. In addition, to benchmark manufacturable designs against the freeform near upper limit, we evaluate stacked binary multilayer in-couplers with *L* ∈ {1, …, 6}.

### 3.1 Design principle of in-coupler

The in-coupler is designed to diffract each RGB wavelength into a distinct diffraction order such that all colors share the same in-plane propagation angle inside the waveguide. For the high-index glass waveguide used in this work (S-LAH79, *n*_wg_ = 2.0) [66], and a grating period of Λ_*x*_ = 2.0 μm, the diffraction orders are chosen as *m*_R_ = 4, *m*_G_ = 5, and *m*_B_ = 6, satisfying *m*_R_ *λ*_R_ = *m*_G_ *λ*_G_ = *m*_B_ *λ*_B_, which ensures achromatic in-plane propagation and prevents spatial color misalignment. Conventional optical glasses with lower refractive indices limit the guided angular range, restricting FoV. In contrast, high-index glasses such as S-LAH79 expand the TIR window, allowing a wider FoV while maintaining transparency and compatibility with large-area glass processing. The resulting propagation angle *θ*_wg_ satisfies the TIR constraint, $\theta_{\text{wg}}>\theta_{c}=\sin^{-}1\left(n_{\text{air}}/n_{\text{wg}}\right)=30{}^{\circ}$ , ensuring that the coupled light remains guided to reach the out-coupler. To achieve symmetric FoV coverage, the range from −*θ*_inc,min_ to +*θ*_inc,min_ is taken as the effective FoV, where *θ*_inc,min_ is obtained from the grating equation for the selected *θ*_wg_. For the TIR-limited case of *θ*_wg_ = 30°, this corresponds to *θ*_inc,min_ ≈ 20.5°, resulting in a symmetric FoV of approximately 41°.

Since the FoV corresponds to a continuous range of incidence angles, we discretize it into a finite set of sampling angles for optimization. In this work, the range from −*θ*_inc,min_ to +*θ*_inc,min_ is uniformly sampled into 11 incidence angles. Each sampling angle corresponds to a specific field point within the eyebox, and the metasurface is optimized to maximize coupling efficiency into the designated diffraction order for all RGB wavelengths while suppressing power in undesired orders.

To ensure both spectral and angular uniformity, the optimization adopts a multi-objective formulation in which the FoM is evaluated independently for each wavelength-angle pair. An adaptive weighting scheme is employed, updating the weight for each condition dynamically based on its current FoM value, such that sampled incidence angles or wavelengths with lower performance are assigned higher priority, ensuring balanced performance across the entire FoV and spectrum.

The FoM for a given wavelength *λ* and incidence angle *ϕ* is defined as the sum of two normalized terms:

(16) $$FoM\left(\lambda ,\phi \right)=-\left|\frac{P_{m_{\text{out}}}\left(\lambda ,\phi \right)}{P_{\text{dist}}\left(\lambda ,\phi \right)}-1\right|-\left|\frac{P_{m_{\text{out}}}\left(\lambda ,\phi \right)}{P_{\text{total}}\left(\lambda ,\phi \right)}-1\right|,$$

where $P_{m_{\text{out}}}\left(\lambda ,\phi \right)$ denotes the optical power coupled into the designated target diffraction order *m*_out_ for a given wavelength *λ* and sampled incidence angle *ϕ*; $P_{\text{dist}}\left(\lambda ,\phi \right)=\sum_{m\in M_{\text{dist}}}P_{m}\left(\lambda ,\phi \right)$ represents the total power carried by distortion-causing diffraction orders, where $M_{\text{dist}}$ comprises all orders whose in-plane wavevectors satisfy the TIR condition in the waveguide and are therefore capable of reaching the out-coupler and being emitted into the eyebox, thereby producing ghost images or geometric distortions in the perceived virtual image. The angle- and wavelength-dependent bounds that define $M_{\text{dist}}$ (i.e., *m*_min_(*λ*, *ϕ*) ≤ *m* ≤ *m*_max_(*λ*, *ϕ*)) are summarized in Table 1; and *P*_total_(*λ*, *ϕ*) = *∑*_*m*_ *P*_*m*_(*λ*, *ϕ*) denotes the total power summed over all propagating diffraction orders for (*λ*, *ϕ*). In Eq. (16), the ratio $P_{m_{\text{out}}}/P_{\text{dist}}$ quantifies the *purity* of the target order by measuring its power relative only to distortion-causing orders, such that maximizing this ratio corresponds to minimizing the influence of those orders regardless of the power in non-distorting orders. In contrast, the ratio $P_{m_{\text{out}}}/P_{\text{total}}$ quantifies the *efficiency* by measuring the fraction of the total power in all propagating diffraction orders that is coupled into the target order, thereby maximizing the amount of input power directed into the desired order. The negative absolute-value terms impose a penalty on deviations of both purity and efficiency from unity, ensuring that the FoM reaches its maximum when both metrics are simultaneously maximized.

**Table 1:** Minimum and maximum diffraction orders satisfying the TIR condition in the waveguide for each sampled incidence angle *θ*_inc_ and RGB wavelength (using *λ*_*R*_ = 675 nm, *λ*_*G*_ = 540 nm, *λ*_*B*_ = 450 nm).

*θ*_inc_ (deg)	Red		Green		Blue	
	*m*_min_	*m*_max_	*m*_min_	*m*_max_	*m*_min_	*m*_max_
−20	2	4	3	6	3	7
−16	3	5	3	6	4	7
−12	3	5	3	6	4	7
−8	3	5	4	6	4	8
−4	3	5	4	7	5	8
+0	3	5	4	7	5	8
+4	4	6	4	7	5	9
+8	4	6	5	7	6	9
+12	4	6	5	8	6	9
+16	4	6	5	8	6	10
+20	4	6	5	8	6	10

The total FoM, FoM_total_, is obtained by aggregating the contributions from all wavelength-angle pairs, with each pair assigned an adaptive weight *w*(*λ*, *ϕ*) to balance their relative importance during optimization:

(17) $${FoM}_{\text{total}}=\underset{\lambda }{\sum }\underset{\phi }{\sum }w\left(\lambda ,\phi \right)\;FoM\left(\lambda ,\phi \right).$$

### 3.2 In-coupler optimization results

All electromagnetic simulations were performed with the open-source MEEP finite-difference time-domain (FDTD) solver [67], and the adjoint optimization was implemented using Meep’s Python interface. All designs were optimized under a TE polarization condition, consistent with the linearly polarized display light and the guided mode used in AR waveguides. A uniform Yee grid of 50 pixels μm^−1^ (Δ*x* = 20 nm) ensured at least twenty samples per wavelength at the shortest wavelength *λ*_min_ = 450 nm. Perfectly matched layers of thickness 1 μm were applied, while Bloch-periodic boundary conditions were used along *x*. To ensure manufacturability, a conic minimum-feature filter with radius *r*_min_ = 50 nm was applied to the design variables before projection, enforcing a 50 nm minimum linewidth and gap by smoothing the spatial profile and eliminating unrealistically fine features [65]. All computations were run on a 64-core AMD Threadripper PRO 5995WX workstation with 256 GB RAM. A single forward-adjoint pair took approximately 2 min of wall-clock time, and the optimization completed in about 20 h over 600 iterations.

**Figure 5:**
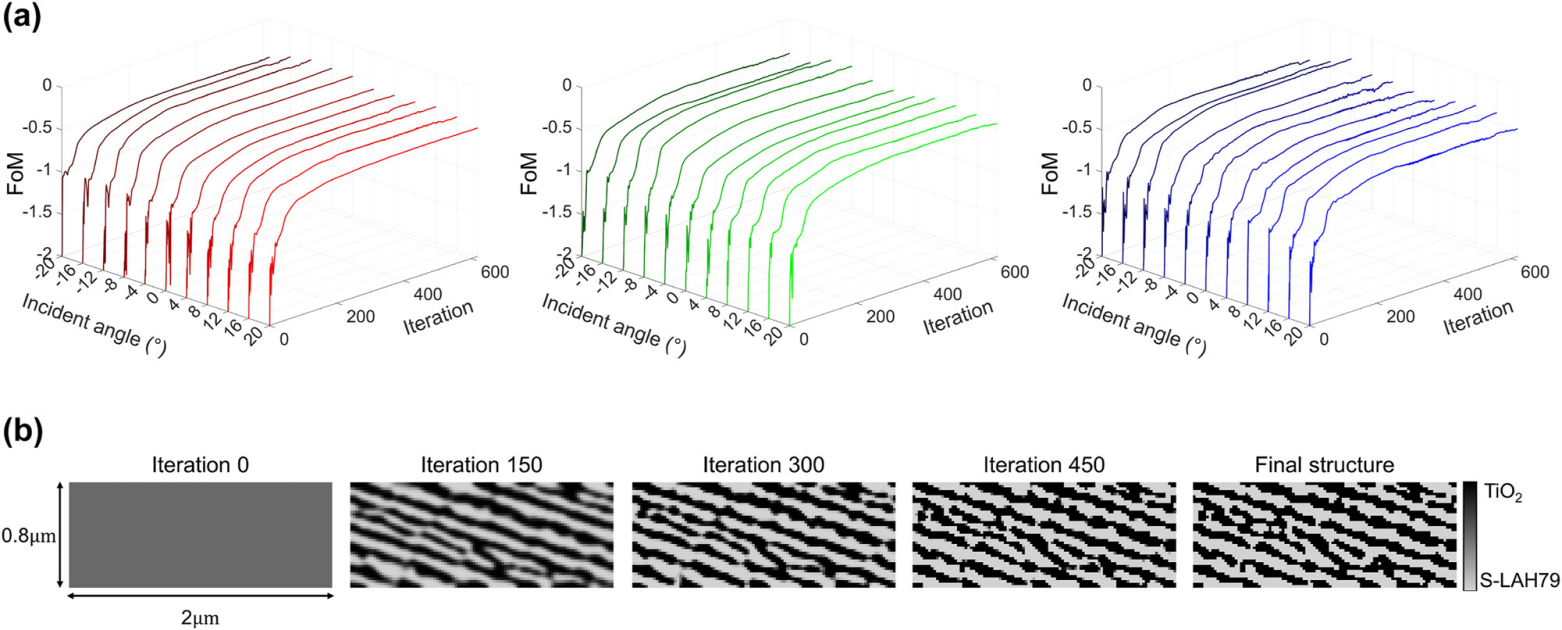
Performance evolution and geometry development of the optimized freeform in-coupler. (a) Evolution of the FoM as a function of iteration and incident angle for the three wavelengths: *λ*_*r*_ (left), *λ*_*g*_ (center), and *λ*_*b*_ (right). The FoM increases uniformly across angles and wavelengths, rapidly improving in the early stages and converging after about 600 iterations. (b) Structural snapshots at selected iterations (0, 150, 300, 450, and final) showing the transition from an initially uniform design to a binary TiO_2_/S-LAH79 structure that efficiently directs power into the designated diffraction orders.

Figure 5 presents the performance evolution and final geometry of the optimized free-form in-coupler. Figure 5(a) shows the evolution of the FoM as a function of iteration number and sampled incidence angle for the three design wavelengths: *λ*_*r*_ (red curves, left), *λ*_*g*_ (green curves, center), and *λ*_*b*_ (blue curves, right). Across all sampled incidence angles and the three design wavelengths, the FoM increases uniformly from its initial value of −2 toward zero, reflecting simultaneous improvements in both purity and efficiency across the entire design space. The FoM evolutions for all design wavelengths exhibit a rapid initial increase, indicating that the adjoint-based optimization efficiently exploits dominant gradient components to achieve substantial improvements in the early stages. As the iteration progresses, the projection parameter *β* gradually increased, driving the design toward a more binary material distribution. The FoM subsequently converges after approximately 600 iterations, indicating that the geometry has stabilized. Figure 5(b) illustrates the structural evolution of the in-coupler during the optimization process. Structural snapshots at selected iterations (0, 150, 300, 450, and final) reveal the gradual transition from a uniform refractive index profile to a binary pattern of TiO_2_ and S-LAH79. Optimization transforms the initially uniform design into a patterned refractive-index distribution that stabilizes into a structure yielding high diffraction efficiency with reduced power in undesired orders.

**Figure 6:**
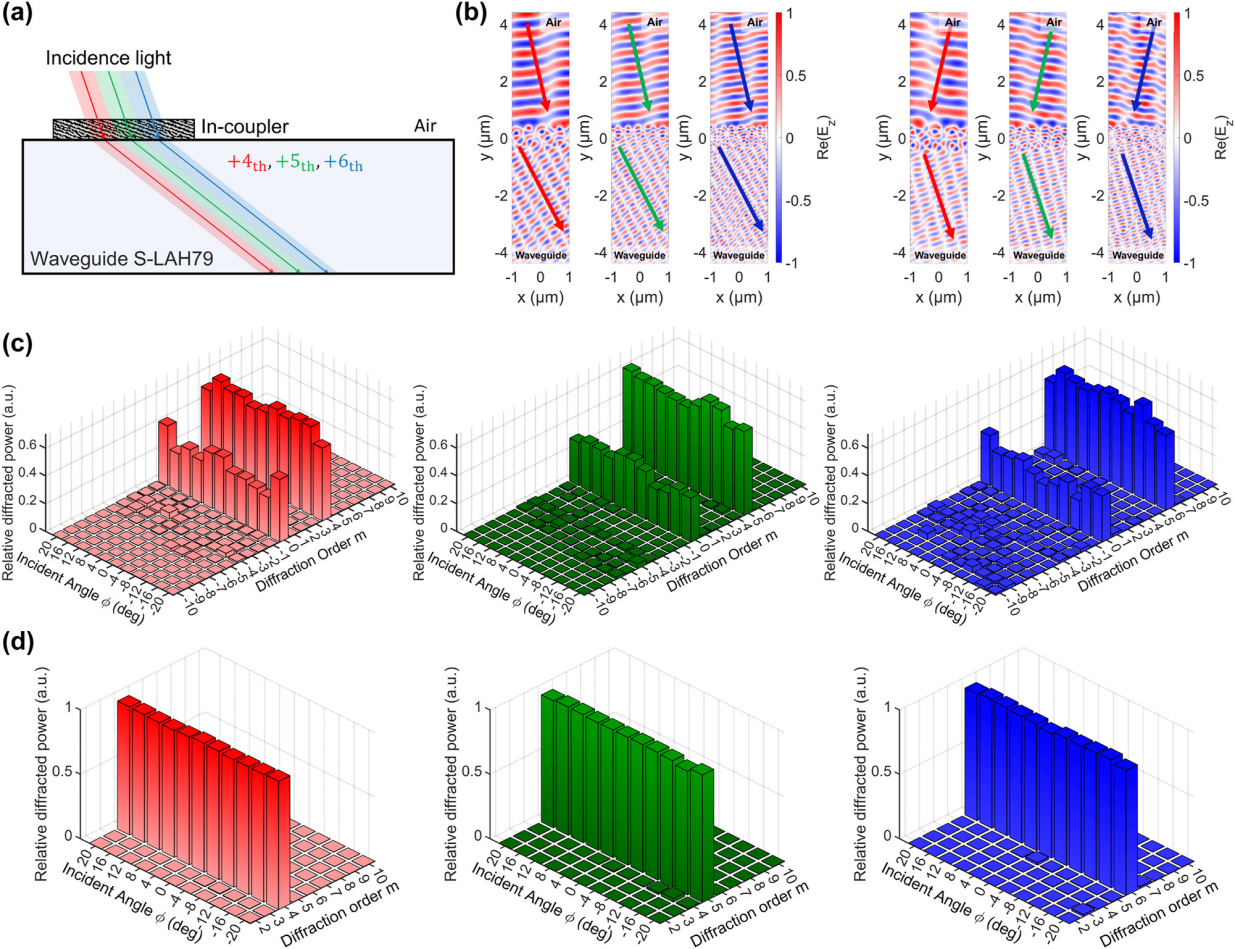
Performance of the optimized freeform in-coupler metasurface. (a) In-coupling geometry: RGB light incident from air is diffracted into the +4th (red), +5th (green), and +6th (blue) diffraction orders, producing a common in-plane guided direction inside the S-LAH79 waveguide. (b) Representative electric-field profiles Re{*E*_*z*_} for two incident angles (*ϕ* = −12° and +12°) across the three design wavelengths, showing dominant coupling into the designated guided orders with minimal residual distortions. (c) Efficiency distribution: fraction of incident power diffracted into each order as a function of incident angle, demonstrating dominant coupling (∼60 %) into the designated orders across all sampled angles. (d) Purity distribution: target-order power normalized by the cumulative power in distortion-causing guided orders, remaining close to unity across angles and confirming selective suppression of unintended guided orders.

RGB light incident from air is diffracted by the in-coupler into the +4th (R), +5th (G), and +6th (B) orders, producing a common in-plane guided direction inside the S-LAH79 waveguide as designed in Section 3.1, as illustrated in Figure 6(a). Figure 6(b) shows representative normalized electric-field profiles, Re{*E*_*z*_}, for two selected incidence angles among the sampled set: *ϕ* = −12° (left) and *ϕ* = +12° (right). For each angle, the field distributions are presented for the three design wavelengths. For each wavelength, the field profiles reveal minor wavefront distortions arising from residual contributions of non-target diffraction orders; however, the dominant portion of the incident wave is diffracted into the designated diffraction order and couples into a guided mode propagating at the same in-plane angle (colored arrows) across all three wavelengths. Figure 6(c) reports the efficiency distribution, i.e., the fraction of the total power in all propagating diffraction orders directed into order *m*_out_ (R: +4, G: +5, B: +6), as a function of sampled incidence angle *ϕ* and diffraction order *m*. For all three wavelengths, the dominant components correspond to the designated diffraction order across the entire incident angular range, with approximately 60 % of the incident power coupled into the target order. A noticeable zeroth-order component is also present; however, this residual zero-order component propagates directly into the air rather than coupling into the waveguide, and thus does not contribute to the guided modes that reach the out-coupler. This observation motivates the analysis of purity in Figure 6(d). Figure 6(d) shows the purity distribution, where the target-order power is normalized by the cumulative power in distortion-causing orders $M_{\text{dist}}$ (i.e., those satisfying the TIR condition and therefore capable of reaching the out-coupler). The relative diffracted power components remain close to unity over all sampled angles, demonstrating that, among the distortion-causing guided orders, diffraction power is concentrated almost exclusively in the target order. This guided-order suppresses unintended diffraction orders, leading to improved visual image quality by reducing ghost images and geometric distortions in the perceived virtual image.

### 3.3 Multilayer in-coupler

To assess a manufacturable architecture against the near-upper-limit performance of the freeform design, we study a stacked binary multilayer in-coupler with *L* ∈ {1, …, 6} layers. Under the same order assignment and the FoM defined in Eqs. (16) and (17), we regard the freeform design as an empirical upper bound and evaluate binary multilayer in-couplers with *L* ∈ {1, …, 6} to quantify the dependence of efficiency and purity on *L*. The case of *L* = 1 corresponds to a single-layer grating benchmark, and its performance directly illustrates the limitations of a simple structure in simultaneously achieving high efficiency and high target-order purity.

Each multilayer design consists of TiO_2_ and S-LAH79 binary pixels with grating period Λ_*x*_ = 2.0 μm along the coupling direction. All layers are in-plane co-registered, each with thickness *t* = 0.3 μm, yielding a total layer thickness of *L t* for *L* ∈ {1, …, 6}. Based on the 2 μm period and the 20 nm spatial discretization, each layer contains 101 independent design parameters. Thus, a six-layer structure (*L* = 6) provides a total of 606 degrees of freedom for optimization. To reflect fabrication limits, we enforce a minimum feature size of 20 nm, which is compatible with advanced electron-beam lithography (EBL). To ensure a fair comparison with the freeform in-coupler, we retain the same order assignment (R: +4, G: +5, B: +6), the same FoM definition in Eqs. (16) and (17), and the same angular sampling of the symmetric FoV, using sampled eleven incidence angles uniformly spaced from *θ*_inc,min_ to +*θ*_inc,min_. For each wavelength-angle pair, diffracted powers are obtained by the plane-wave decomposition of Section 3.1, and adaptive weights are applied to balance uniformity.

**Figure 7:**
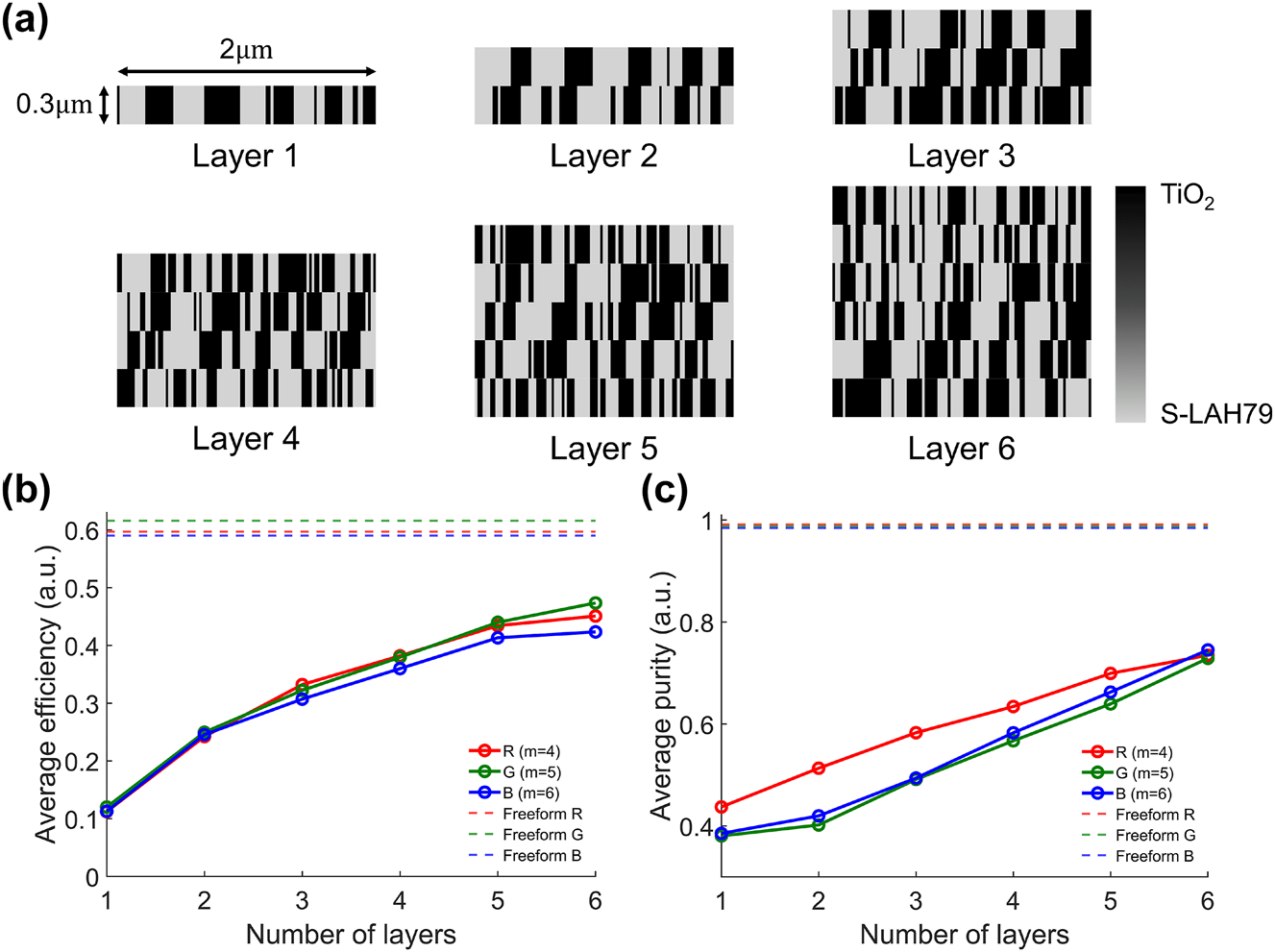
Performance of binary multilayer in-couplers compared with the freeform design. (a) Optimized TiO_2_/S-LAH79 binary structures for *L* = 1–6 layers, designed with a 2 μm and 0.3 μm layer thickness. Additional layers increase optical thickness and degrees of freedom, enabling finer phase control. (b) Angle-averaged efficiency into the designated diffraction orders (*m*_R_ = +4, *m*_G_ = +5, *m*_B_ = +6) versus the number of layers *L*. Efficiency rises monotonically with *L*, with rapid gains up to *L* ≈ 3 and diminishing improvements thereafter. The freeform design (dashed lines) serves as an empirical upper bound, above the *L* = 6 multilayer case. (c) Angle-averaged purity increases steadily with *L* without saturation up to *L* = 6. This observation indicates that added layers primarily suppress non-target guided orders, thereby reducing ghosting and geometric distortions in the visualized image.

The optimized binary TiO_2_/S-LAH79 designs for *L* ∈ 1, …, 6 are shown in Figure 7(a), and as *L* increases, the added layers and optical thickness provide extra degrees of freedom, enabling finer phase control and thus enhancing coupling into the designated diffraction orders across the FoV-sampled incidence angles.

Figure 7(b) shows the angle-averaged target-order efficiency as a function of the number of layers *L*. The efficiency is defined as the fraction of the total transmitted diffracted power directed into the designated order *m*_out_, averaged over the eleven uniformly sampled incidence angles. This plot clearly quantifies the poor performance of the *L* = 1 benchmark, which yields an average efficiency of only ≈12–15 %. The curves increase monotonically with *L*, with a pronounced gain from *L* = 1 to *L* ≈ 3 and progressively smaller increments thereafter. At each layer count *L*, the R/G/B curves are nearly coincident, indicating that uniformity is preserved for every *L*. A dashed line marks the empirical upper bound set by the freeform design; although efficiency continues to improve with *L*, the gains are modest beyond *L* ≈ 5 and the *L* = 6 case remains below the freeform performance. Figure 7(c) presents the angle-averaged purity, defined in Eq. (16) as the target-order power normalized by the cumulative power in distortion-causing guided orders, versus the number of layers *L*. The limitation of the simple grating is even more distinct in this metric, with the *L* = 1 benchmark showing an average purity below 40 %. Purity increases monotonically with *L* and shows no clear saturation up to *L* = 6, in contrast to the target-order efficiency which exhibits diminishing gains beyond *L* ≈ 3. This behavior indicates that added layers primarily suppress distortion-causing guided orders, reducing *P*_dist_ in Eq. (16), rather than increasing the designated-order power $P_{m_{\text{out}}}$ . Consequently, purity continues to improve even when the efficiency increment is small, which reduces ghosting and geometric distortion in the perceived image. These trends confirm that additional layers provide essential design freedom to suppress distortion-causing guided orders and improve efficiency and purity beyond what is achievable with the single-layer grating.

## 4 Out-coupler design and optimization

The out-coupler plays a central role in determining the overall performance of the waveguide display by directing guided modes into the designated transmission orders, preserving residual power for eyebox expansion, and maintaining optical see-through in the presence of ambient light. Unlike the in-coupler, the out-coupler must simultaneously satisfy multiple objectives: high-efficiency transmission into the assigned diffraction orders, strong TIR-preserved reflection to redirect guided power, and suppression of higher-order diffraction under normal-incidence external light. This section presents the design principles of the out-coupler, the optimization framework and results for the freeform case, an analysis of binary multilayer structures benchmarked against the freeform upper bound, and the imaging performance evaluation in both frequency and image domains.

### 4.1 Design principle of out-coupler

The out-coupler diffracts the guided modes introduced by the in-coupler into the opposite diffraction orders, ensuring that each incident light angle is mapped to its corresponding transmitted light angle, thereby avoiding angular distortion in the reconstructed virtual image. For the out-coupler, the grating period was set to Λ_*x*_ = 2.0 μm, identical to that of the in-coupler, since both components rely on the same phase-matching condition to couple the designated diffraction orders and the grating height was chosen as 0.8 μm. The out-coupler is optimized over the same set of design wavelengths as the in-coupler, while angular sampling is performed across the guided in-plane propagation angles generated by the in-coupler. All guided angles lie within the TIR window [30°, 60°]; for optimization, this was uniformly discretized into 11 angles, which constitute the angular sampling set for the out-coupler.

For each pair (*λ*, *θ*_wg_), the diffracted power in every order is evaluated using the plane-wave decomposition of Section 3.1. We denote by $P_{m}^{\text{tr}}\left(\lambda ,\theta_{\text{wg}}\right)$ and $P_{m}^{\text{re}}\left(\lambda ,\theta_{\text{wg}}\right)$ the transmitted and reflected powers, respectively, into diffraction order *m* at the out-coupler. In addition, to characterize the response to normally incident external illumination, we consider a TE-polarized plane wave incident from air (*ϕ* = 0) and define $P_{0}^{\text{ext}}\left(\lambda \right)$ as the transmitted power into the zeroth order under this condition. For transmission, the target orders are *m*_out_(*λ*) ∈ {−4, −5, −6} for R, G, and B, respectively. For reflection, we quantify the TIR-preserved component as $P_{0}^{\text{re}}\left(\lambda ,\theta_{\text{wg}}\right)$ , defined as the zero-order term that remains guided within the waveguide.

To achieve a specified power split between transmission into the designated orders and TIR-preserved reflection for eye-box expansion, we define two complementary allocation objectives. The transmission objective aims to achieve a fraction *α*_T_ = 0.15 of the total diffracted power in the designated transmission order:

(18) $$\begin{array}{cc} & {FoM}_{\text{T}}\left(\lambda ,\theta_{\text{wg}}\right)\\ & \;=-\left|\frac{P_{m_{\text{out}}\left(\lambda \right)}^{\text{tr}}\left(\lambda ,\theta_{\text{wg}}\right)}{\underset{m}{\sum }P_{m}^{\text{tr}}\left(\lambda ,\theta_{\text{wg}}\right)+\underset{m}{\sum }P_{m}^{\text{re}}\left(\lambda ,\theta_{\text{wg}}\right)}-\alpha_{\text{T}}\right|. \end{array}$$

The reflection objective targets *α*_R_ = 0.85 in the TIR-preserved (zero-order) reflection:

(19) $$\begin{array}{cc} & {FoM}_{\text{R}}\left(\lambda ,\theta_{\text{wg}}\right)\\ & \;=-\left|\frac{P_{0}^{\text{re}}\left(\lambda ,\theta_{\text{wg}}\right)}{\underset{m}{\sum }P_{m}^{\text{tr}}\left(\lambda ,\theta_{\text{wg}}\right)+\underset{m}{\sum }P_{m}^{\text{re}}\left(\lambda ,\theta_{\text{wg}}\right)}-\alpha_{\text{R}}\right|. \end{array}$$

To preserve optical see-through and suppress nonzero-order diffraction of normally incident external light, we impose an see-through view objective that maximizes zeroth-order transmission:

(20) $${FoM}_{\text{ext}}\left(\lambda \right)=-\left|\frac{P_{0}^{\text{ext}}\left(\lambda \right)}{\underset{m}{\sum }P_{m}^{\text{ext}}\left(\lambda \right)}-1\right|,$$

where $P_{m}^{\text{ext}}\left(\lambda \right)$ denotes the transmitted power into order *m* under normal-incidence external light.

The total out-coupler objective aggregates transmission and reflection over all wavelength and guided angle conditions with independent adaptive weights, and adds the external-light term per wavelength:

(21) $$\begin{array}{cc} & {FoM}_{\text{out}}^{\text{total}}\\ & \;=\underset{\lambda }{\sum }\underset{\theta_{\text{wg}}}{\sum }\left[w_{\text{tr}}\left.\left(\lambda ,\theta_{\text{wg}}\right)\right\}\;{FoM}_{\text{T}}\left(\lambda ,\theta_{\text{wg}}\right)\right.\\ & \;\;\left.\left.+w_{\text{re}}\left(\lambda ,\theta_{\text{wg}}\right)\right\}\;{FoM}_{\text{R}}\left(\lambda ,\theta_{\text{wg}}\right)\right]\\ & \;\;+\underset{\lambda }{\sum }w_{\text{ex}}\left(\lambda \right)\;{FoM}_{\text{ext}}\left(\lambda \right), \end{array}$$

where *w*_tr_, *w*_re_, and *w*_ex_ are adaptive weights updated during optimization to emphasize underperforming wavelength-angle conditions.

### 4.2 Out-coupler optimization results

All electromagnetic simulations for the out-coupler used the same numerical settings as the in-coupler. Differences were limited to the source conditions, the monitor placements, and the FoM used for optimization. The runtimes were ≈5 min per forward-adjoint pair and ≈50 h for an optimization of over 600 iterations.

**Figure 8:**
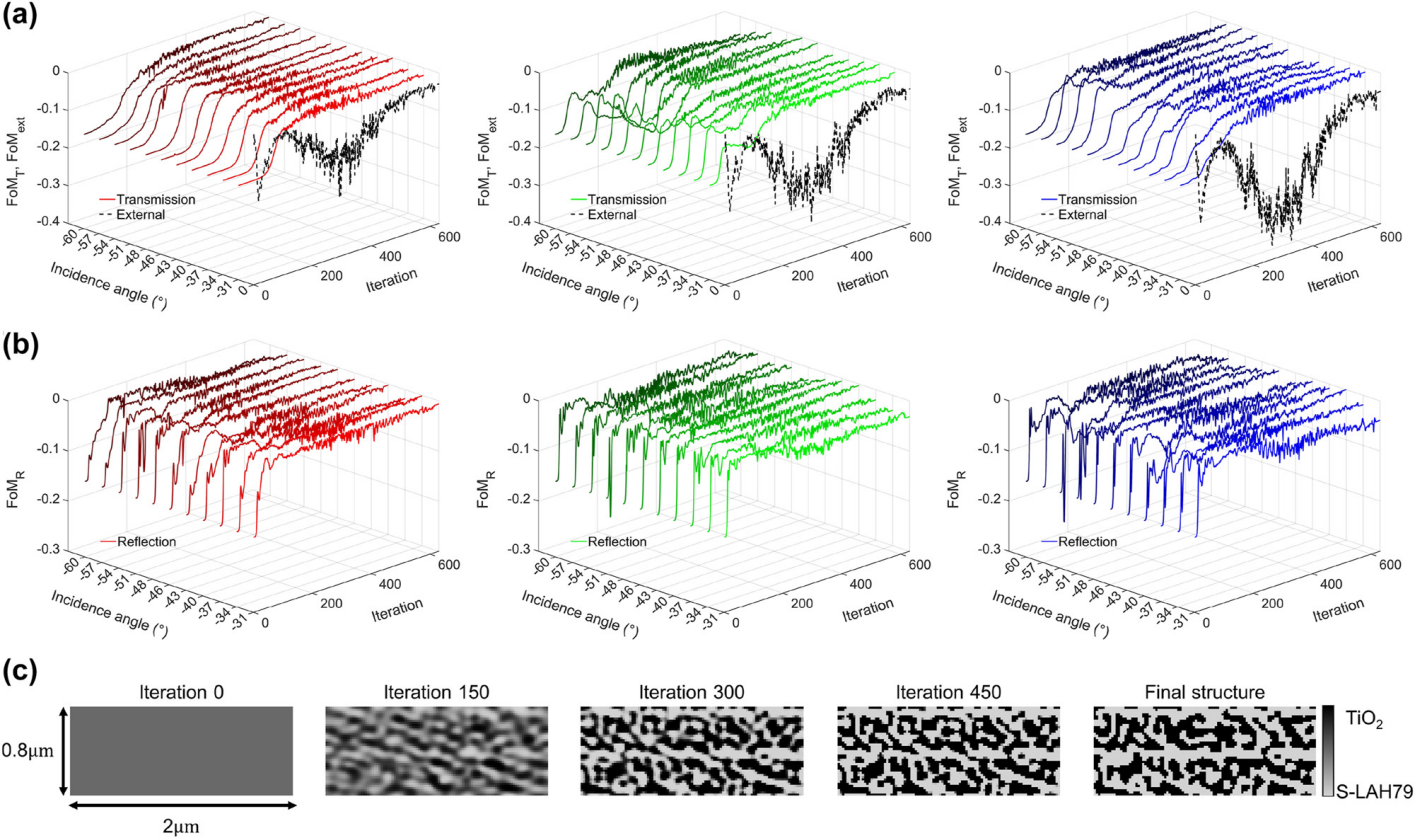
Performance evolution and geometry development of the optimized freeform out-coupler. (a) Evolution of the transmission objective (FoM_T_, solid curves) and the see-through view objective (FoM_ext_, dashed curves) as functions of iteration and guided incidence angle for R, G, and B. (b) Evolution of the reflection objective (FoM_R_) across the sampled guided-angle set. In both cases, the objectives converge toward zero, indicating attainment of the target power split: *α*_T_ = 0.15 into the designated transmission orders and *α*_R_ = 0.85 into the TIR-preserved zeroth-order reflection, together with maximized zeroth-order transmission for normal-incidence external light. (c) Structural snapshots during optimization at selected iterations (0, 150, 300, 450, and final). The initially uniform medium gradually evolves into a binary TiO_2_/S-LAH79 geometry that supports phase matching to couple guided modes into the target diffraction orders while returning residual guided power as *m* = 0 TIR; simultaneously, the structure is designed to transmit normally incident ambient light in the zeroth order, suppressing higher-order diffraction.

The iterative evolution of the transmission, reflection, and see-through scene objectives across the sampled guided-angle set, along with structural snapshots that illustrate the binarization of the freeform out-coupler, is shown in Figure 8. In Figure 8(a), the solid curves show the transmission objective FoM_T_ and the dashed curves show the see-through view objective FoM_ext_ as functions of iteration and the sampled guided angles. For all three design wavelengths, FoM_T_ increases from its initial negative value toward zero across the full angular range, indicating that the fraction of power directed into the designated out-coupling orders approaches the target *α*_T_ = 0.15. The external-light term FoM_ext_ shows a brief dip at intermediate iterations but returns toward zero as the design converges. At the final iteration, the average transmission objective over all wavelengths and guided angles is ${\bar{FoM}}_{\text{T}}=-0.0154$ , and the see-through view objective averaged over RGB at normal incidence is ${\bar{FoM}}_{\text{ext}}=-0.0399$ .

The reflection objective FoM_R_ over the same sampled guided-angle set is shown in Figure 8(b). A rapid early improvement is followed by gradual convergence toward zero for all wavelengths and angles, indicating that the TIR-preserved reflection fraction approaches the target *α*_R_ = 0.85 uniformly across the sampled guided-angle set. Quantitatively, the average reflection objective over all wavelengths and sampled guided angles at the final iteration is ${\bar{FoM}}_{\text{R}}=-0.0140$ . Overall, the results in Figure 8(a) and (b) confirm that the out-coupler achieves the intended transmission-reflection split while enforcing zeroth-order transmission for normally incident external light.

Structural snapshots of the freeform geometry at iterations 0, 150, 300, 450, and at the final design are shown in Figure 8(c). Starting from a uniform refractive index distribution, the structure gradually evolves through progressive binarization during optimization into a well-defined binary pattern of TiO_2_ and S-LAH79. The resulting pattern furnishes the spatial Fourier content required to phase-match the target orders (*m*_*R*_ = −4, *m*_*G*_ = −5, *m*_*B*_ = −6) while returning the residual power to TIR, in agreement with the near-zero residuals of FoM_T_, FoM_R_, and FoM_ext_ at convergence. The final structure provides the index modulation necessary to direct a controlled fraction of guided power into the target out-coupling orders while returning the remainder to TIR, in agreement with the objectives quantified by FoM_T_ and FoM_R_.

**Figure 9:**
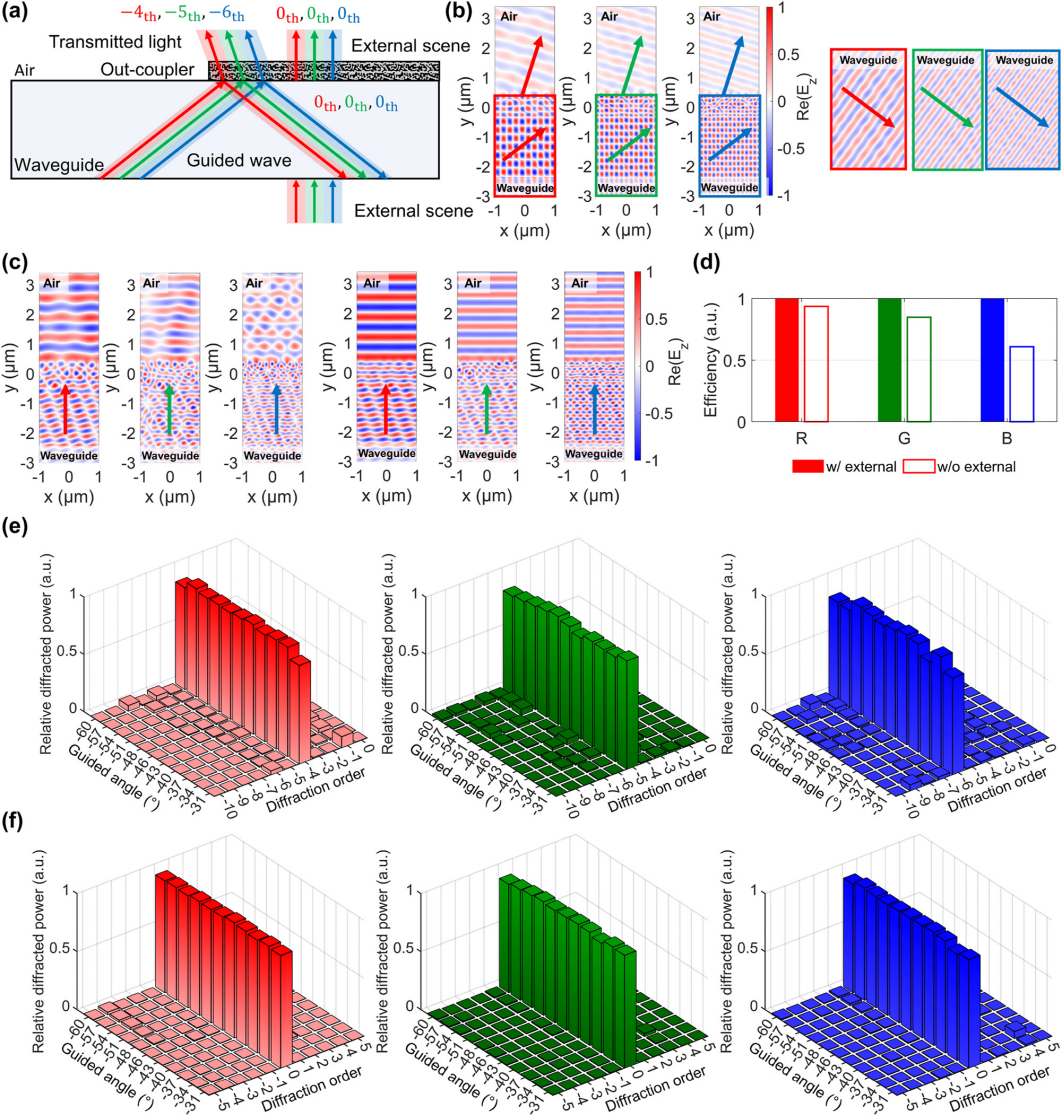
Performance of the optimized freeform out-coupler. (a) Operating principle: guided waves are diffracted into the designated transmission orders (−4, −5, −6) for RGB light, while residual power is returned to the waveguide by TIR-preserved reflection, and the see-through scene is transmitted in the zeroth order to maintain see-through. (b) Electric-field distributions for guided incidence at *θ*_wg_ = −55°: transmission into the designated orders at a common emission angle of *θ*_out_ ≈ +16.7°, and zeroth-order guided reflection preserving the input angle. (c) Comparison of external-light response without (left) and with (right) the see-through scene objective. Without the see-through scene objective, normal-incidence light is redistributed into multiple nonzero diffraction orders, distorting the scene. With the see-through scene objective, higher-order content is suppressed and power is confined near the zeroth order, preserving see-through. (d) Zeroth-order transmission efficiency under normal-incidence external light, with and without the see-through scene objective. With the see-through scene objective, zeroth-order transmission exceeds 99 % for all RGB channels; without it, the zeroth-order transmission drops to ≈90 % at red and to ≈60 % at blue, as power is redistributed into higher diffraction orders. (e) Transmission efficiency: across all sampled angles, the designated out-coupling orders dominate, with normalized transmission >90 % for RGB. (f) Reflection efficiency: reflected power is concentrated in the zeroth order, with normalized reflection >95 % across wavelengths, confirming that residual guided power is returned by TIR without generating parasitic orders.

The operating principle of the out-coupler is summarized in Figure 9(a): guided waves are directed into the opposite diffraction orders (*m*_*R*_ = −4, *m*_*G*_ = −5, *m*_*B*_ = −6) for transmission to air, residual guided power is returned as specular (*m* = 0) reflection to support eyebox expansion, and normally incident external light is transmitted in the zeroth order to preserve see-through. Figure 9(b) shows electric-field distributions of transmission to air and guided reflection for a guided incidence of *θ*_wg_ = −55°, one of the eleven guided angles used in the optimization. In transmission to air, red, green, and blue are predominantly directed into the prescribed out-coupling orders (−4, −5, −6), with a common emission angle of *θ*_out_ ≈ +16.7° for all three wavelengths, consistent with the designed order-angle correspondence. In guided reflection, the same incidence is returned in the zeroth order and continues to propagate at +55° inside the waveguide, as targeted by the TIR-preserved reflection objective.

The response to normal-incidence external light is compared in Figure 9(c) for two designs, one optimized without the see-through scene objective and the other optimized with it. When the see-through scene objective is omitted, the periodic out-coupler behaves as a strong diffractive element that broadly redistributes normally incident scene light into multiple nonzero orders, instead of transmitting it in the zeroth order. The resulting uncontrolled higher-order content causes off-normal propagation and angular spread, which in turn produces geometric distortion of the real scene. When the see-through scene objective is included in the optimization, higher-order transmission is suppressed and power is confined near the zeroth order, yielding straight-through, achromatic see-through under normal incidence. The corresponding quantitative comparison in Figure 9(d) shows that, when the see-through scene objective is included, the zeroth-order transmission at normal incidence exceeds 99 % of the transmitted power for R, G, and B. Without this objective, the zeroth-order share is substantially reduced and power is redistributed into nonzero diffraction orders: under normal incidence, the red channel remains near 90 % in the zeroth order, whereas the blue channel drops to the low −60 % range, consistent with the field distributions in Figure 9(c).

Transmission efficiency as a function of guided angle, resolved by diffraction order, is plotted in Figure 9(e). For each sampled *θ*_wg_ and for each of the three wavelengths, the normalized transmitted power is shown for each diffraction order *m*. Across the guided-angle set, the designated out-coupling orders dominate, whereas non-target transmission orders remain nearly zero over all sampled angles, with only minor residuals. Averaged over the eleven guided angles, the normalized transmitted power in the designated order exceeds 90 % for R, G, and B, indicating that the vast majority of the total transmitted power is directed into the intended order.

Reflection efficiency as a function of guided angle, resolved by diffraction order, is plotted in Figure 9(f). For each sampled *θ*_wg_ and wavelength, the normalized reflected power is shown for each diffraction order *m*. Across the guided-angle set, the reflected power is overwhelmingly concentrated in the zeroth order, whereas higher-order guided reflections are negligible over all angles. Averaged over the sampled guided angles, the normalized reflected power in the zeroth order exceeds 95 % for R, G, and B, confirming that the residual power is returned to the waveguide by TIR without generating parasitic guided orders, thereby supporting eyebox expansion and limiting ghost paths at the out-coupler, in line with the reflection objective. This TIR-preserved reflection mechanism allows the residual guided power to undergo multiple internal reflections and be progressively extracted at successive positions, thereby enabling natural pupil replication along the propagation direction and supporting eyebox expansion.

### 4.3 Multilayer out-coupler

To assess a manufacturable architecture against the freeform upper bound, we investigate binary multilayer out-couplers with *L* ∈ {1, …, 6} layers and benchmark them against the unconstrained design. Each multilayer comprises TiO_2_ and S-LAH79 binary pixels with grating period Λ_*x*_ = 2.0 μm, identical to the in-coupler. All layers are laterally aligned, each with thickness *t* = 0.3 μm, giving a total thickness *L*_*t*_. To reflect fabrication limits, a minimum feature width of 20 nm is enforced, consistent with the in-coupler design and EBL capabilities. Unlike the freeform design, the multilayer optimization employs an efficiency-based objective, where efficiency is defined as the power in a specified diffraction order normalized by the total diffracted power. For transmission, we maximize efficiency into the assigned target orders (red: −4, green: −5, blue: −6). For guided-mode reflection, we maximize the zeroth-order efficiency. For normal-incidence see-through, we maximize the zeroth-order transmission. The overall FoM is a weighted sum of these three terms, with the weights adaptively adjusted during the optimization.

**Figure 10:**
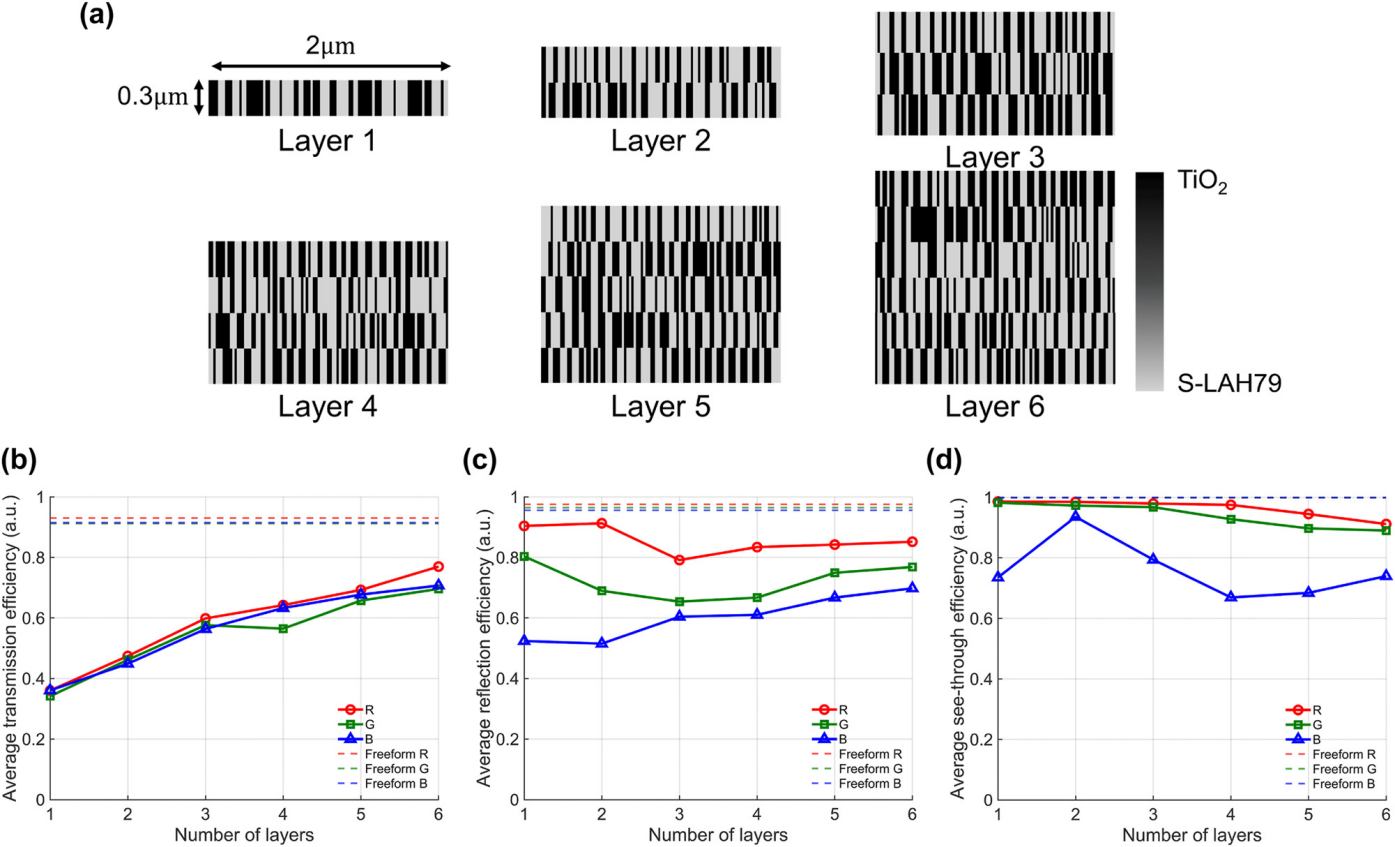
Performance of binary multilayer out-couplers compared with the freeform design. (a) Optimized TiO_2_/S-LAH79 binary structures for *L* = 1–6 layers, designed with a 2 μm and 0.3 μm layer thickness. Additional layers increase optical thickness and degrees of freedom, enabling finer phase control. (b) Angle-averaged transmission efficiencies into the target diffraction orders (R: −4, G: −5, B: −6); dashed curves indicate the freeform empirical upper bounds. Transmission rises monotonically with *L*, with the largest gains from *L* = 1 to *L* = 3. (c) Angle-averaged guided-mode reflection efficiencies; dashed curves denote the freeform reference. Reflection decreases at small *L* as transmission improves, then recovers and increases with additional layers, approaching the freeform level by *L* = 6. (d) Angle-averaged see-through efficiencies; dashed curves indicate the freeform references. At *L* = 1, B is notably lower than R and G; as *L* increases, parasitic higher orders are suppressed and see-through approaches the upper bound. A channel-dependent reduction occurs at small *L* (small for R/G, pronounced for B), after which B increases from *L* ≥ 4 while R/G change only slightly (G nearly unchanged from *L* = 5 to *L* = 6). Taken together, increasing *L* adds design degrees of freedom and progressively narrows the gap to the freeform bound across transmission, reflection, and see-through.

The optimized binary layouts for *L* ∈ {1, …, 6} layers are presented in Figure 10(a). As expected, the *L* = 1 benchmark shows unacceptably low transmission efficiency for the virtual image. As *L* increases, the design evolves from a predominantly one-dimensional single-layer layout to multi-layer configurations that introduce additional degrees of freedom, enabling more precise in-plane modulation of phase and amplitude. The average transmission efficiencies into the designated diffraction orders are plotted in Figure 10(b), where the dashed lines indicate the empirical upper bounds obtained from the freeform design. As the number of layers increases, the R/G/B curves all show a monotonic rise, with the most significant gains observed from one to three layers and more gradual improvements thereafter. This behavior reflects the additional degrees of freedom as the number of layers grows, which allow more effective redistribution of power into the target diffraction orders. The corresponding guided-mode reflection efficiencies are shown in Figure 10(c), where the dashed lines denote the freeform reference. Reflection initially decreases as transmission improves, indicating a trade-off between the two objectives at small *L*. However, with further increase in the number of layers, the reflection efficiencies for all three wavelengths recover and eventually rise, approaching the freeform upper bound at six layers. These results indicate that the multilayer design progressively mitigates the trade-off, enabling high transmission while maintaining strong guided-mode reflection. In particular, the *L* = 1 case serves as a single-layer grating baseline and exhibits limited ability to balance transmission, guided-mode reflection, and see-through efficiency, whereas increasing *L* progressively relaxes these trade-offs, demonstrating the practical necessity of the multilayer architecture for realizing the proposed triple-function out-coupler.

The average see-through efficiencies for normally incident ambient light are shown in Figure 10(d), with the dashed lines indicating the freeform references. At *L* = 1, the blue channel is notably lower than red and green, consistent with stronger higher-order leakage at shorter wavelengths. As *L* increases, parasitic diffraction is suppressed and the see-through efficiency moves closer to the upper bound. At small *L*, the see-through efficiency decreases simultaneously with the increase in transmission efficiency, indicating a transient trade-off; the reduction is small for R and G but pronounced for B. As *L* increases, the blue channel begins to recover from *L* ≥ 4, while R and G continue to decrease slightly. Between *L* = 5 and *L* = 6, the green channel remains nearly unchanged. These results indicate that additional layers will lead to a faster increase in B and a gradual convergence of R toward the freeform upper bound, with G remaining close to that bound as *L* increases. This improvement in see-through efficiency for normal-incidence ambient light is achieved by redistributing power toward the zeroth order through interference among the stacked layers.

### 4.4 Imaging performance

We assess the imaging performances of the see-through scene and the virtual image using the PSF and the MTf. The PSF, *h*(*x*, *y*), is the impulse response of the optics in the image plane, where (*x*, *y*) denote the lateral spatial coordinates. It describes how energy from an ideal point source spreads due to diffraction and residual aberrations. The MTF is defined as the magnitude of the two-dimensional Fourier transform of the PSF as follows:

(22) $$MTF\left(f_{x},f_{y}\right)=\left|F\left\{h\left(x,y\right)\right\}\right|.$$

Under a linear imaging system, the output image is obtained via spatial convolution,

(23) o(x,y)=i(x,y)∗h(x,y),

where *i*(*x*, *y*) is the input image, *o*(*x*, *y*) is the output image, and ∗ denotes 2-D convolution.

**Figure 11:**
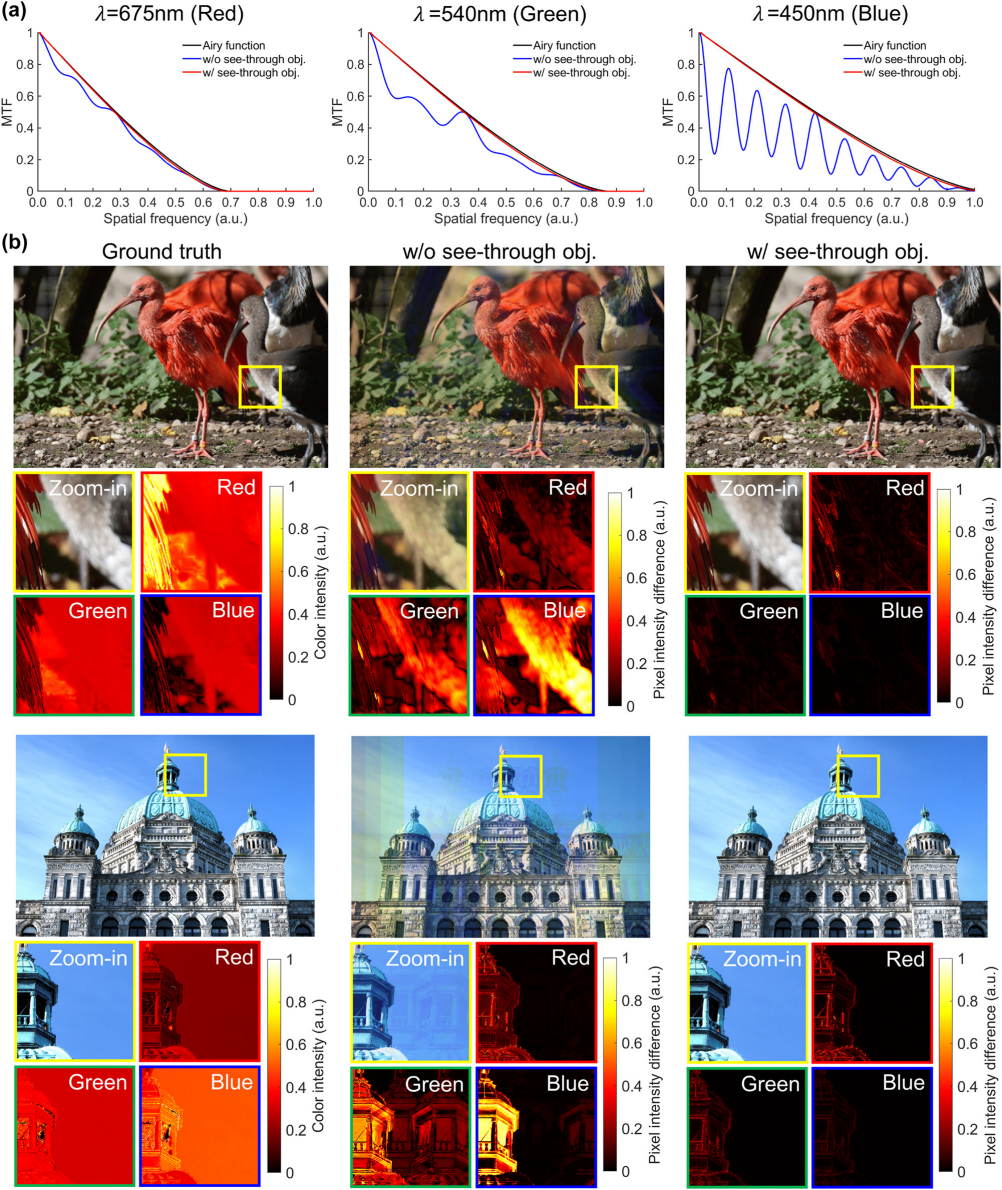
See-through scene imaging performance evaluation of the proposed metasurface out-coupler. (a) MTF curves at *λ* = 675, 540, 450 nm under different conditions: diffraction-limited Airy reference (black), without see-through scene objective (blue), and with see-through scene objective (red). The optimized design closely follows the diffraction limit, whereas the non-optimized design shows early roll-off and ripple artifacts, most severe at 450 nm due to increased coupling into higher diffraction orders. (b) Image-based evaluation on natural (top) and architectural (bottom) scenes. From left to right: ground truth with zoomed-in regions and normalized RGB intensity maps; results without the see-through objective showing blurred edges, ghost artifacts, and strong per-channel pixel-wise differences; results with the see-through objective, where higher-order leakage is suppressed and the mean normalized RGB difference drops from ≈0.126 to ≈0.021 , yielding strong consistency with the ground truth and improved see-through clarity.

The MTF curves for three wavelengths (675, 540, 450 nm) under two cases, without see-through scene optimization and with see-through scene optimization, are presented in Figure 11(a). The spatial frequency axis is normalized to the diffraction-limited cutoff for each wavelength. With see-through scene optimization (red), the MTF closely tracks the diffraction-limited Airy reference across all three wavelengths, indicating robust transfer of mid-high spatial frequencies. In contrast, without this objective (blue), the MTF exhibits early roll-off and pronounced ripple behavior characteristic of undesired higher-order diffraction from the periodic coupler. The degradation intensifies toward shorter wavelengths where the normalized spatial frequency increases, and a larger set of propagating diffraction orders diverts power from the desired order into multiple higher orders. These results show that incorporating the see-through scene term into the optimization objective concentrates power into the zeroth order at normal incidence and suppresses nonzero orders, thereby preserving spatial-frequency bandwidth and contrast. Figure 11(b) extends the evaluation by comparing ground truth scenes with the output results obtained without and with the see-through optimization, including both the reconstructed images and the pixel-wise intensity maps across the RGB channels. The ground truth scenes are shown together with the corresponding normalized RGB intensity distributions for the highlighted regions, which serve as references. Without see-through scene optimization, the output scenes exhibit strong deviations from these references, including blurred edges, ghost artifacts, and pronounced intensity mismatches across the RGB channels, as captured in the pixel-wise difference maps, per-channel absolute intensity difference from the ground truth normalized to [0,1]. With the see-through optimization, higher-order leakage is strongly suppressed and the pixel-wise intensity differences are significantly reduced. Averaged over the RGB channels, the mean normalized deviation decreases from ≈0.126 without optimization to ≈0.021 with optimization. As a result, the output scenes exhibit enhanced see-through clarity with substantially reduced ambient light-induced blur and ghost artifacts.

**Figure 12:**
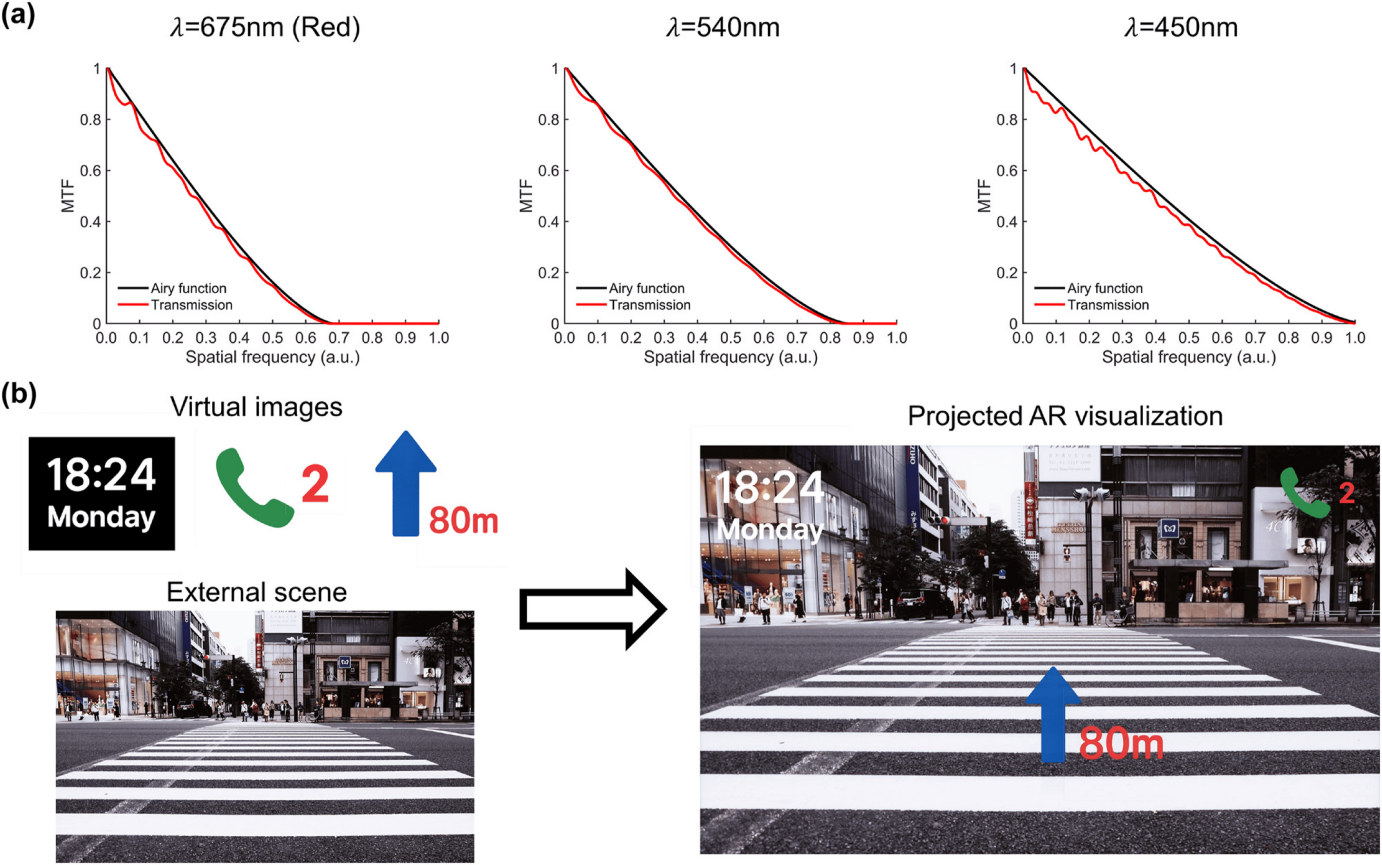
Virtual-image imaging performance. (a) MTF of the virtual image guided through the waveguide (red) compared to the diffraction-limited reference (black) at *λ* = 675, 540, 450 nm and *θ*_wg_ = −49°. Across the visible spectrum, the designed couplers maintain near-diffraction-limited performance with only minor degradation. (b) Projected AR visualization. The AR system output (right) closely matches the superimposed ground-truth virtual and see-through scenes (left), confirming preserved spatial accuracy and fine detail in the virtual overlays while maintaining clarity of the external scene.

The imaging performance of the output virtual image relative to the diffraction limit, along with the overlay visualization that combines the output virtual image and the see-through scene, is illustrated in Figure 12. In Figure 12(a), the MTF is plotted for three representative wavelengths at the guided angle of −49°, corresponding to one of the TIR propagation paths in the waveguide. The red traces denote the MTF of the virtual image guided through the waveguide, while the black curves denote the diffraction-limited reference. Across the visible spectrum, the obtained MTF closely follows the diffraction limit with only modest degradation, indicating that the designed couplers preserve the high-frequency detail of the output virtual image. Figure 12(b) shows the visual outcome of the AR system obtained by superimposing virtual content onto the see-through scene. The ground-truth virtual and see-through views are presented separately on the left, and the combined AR visualization is shown on the right. The results confirm that the optical system preserves the spatial accuracy of the output virtual image, ensuring that virtual overlays are presented without geometric distortion or loss of detail.

Overall, these results demonstrate that explicitly accounting for both the see-through scene and the output virtual image in the optimization framework is essential to achieve high-quality AR performance. The frequency-domain analysis confirms suppression of parasitic diffraction and near-diffraction-limited transfer of high spatial frequencies, while the image-domain evaluations validate the preservation of visual detail and the faithful integration of virtual overlays with real-world scenes. The preservation of both virtual image and the see-through scene quality demonstrates the need to optimize them jointly, ensuring that AR waveguide systems deliver accurate, undistorted virtual images without compromising the clarity of the surrounding environment.

## 5 Conclusions

We have proposed and demonstrated adjoint-based, inverse-designed metasurface in- and out-couplers that address long-standing limitations of waveguide AR – chromatic dispersion, limited eyebox, ghost images, and distortion of the see-through scene. The in-coupler assigns distinct diffraction orders to R/G/B while enforcing a common in-plane propagation angle, thereby achieving achromatic guidance and balanced efficiencies across the visible spectrum. Over 60 % of the incident power is directed into the designated orders across the field of view, with near-unity purity relative to parasitic guided modes.

For the out-coupler, a three-term adjoint objective (designated transmission orders, zeroth-order guided reflection via TIR, and zeroth-order see-through transmission) simultaneously expands the eyebox and preserves see-through clarity. Without the see-through objective, leakage into higher diffraction orders reaches ∼10 % (red) and ∼40 % (blue), producing blur and ghosting; with the objective included, zeroth-order transmission is ≥99 % across RGB and higher-order leakage is <1 % (a 10–40× reduction), while angle-averaged transmission to the image orders exceeds 90 % and zeroth-order guided reflection exceeds 95 %. PSF/MTF analyses confirm near-diffraction-limited quality for both the see-through scene and the virtual image. Finally, fabrication-constrained multilayer metasurfaces show monotonic efficiency gains and approach freeform upper bounds, with diminishing returns beyond five layers. Overall, these results provide a practical route to compact, high-quality AR waveguides and a generalizable design methodology for multifunctional metasurface components.

The present study focuses on unit-cell optimization, but the same framework can be extended to system-level design of spatially varying out-couplers. Such an extension would enable uniform pupil replication and brightness across the eyebox, representing an important next step toward practical AR waveguide systems. In addition, future extensions may incorporate oblique external illumination to evaluate the angular dependence of see-through performance. We also note that the device exhibits polarization-dependent behavior, and a detailed investigation of the orthogonal polarization and unpolarized ambient illumination will be an important direction for future research.
